# Dual-domain engineered exosome-based self-powered microneedle delivery platform for the treatment of infected wounds

**DOI:** 10.1016/j.mtbio.2025.102383

**Published:** 2025-10-07

**Authors:** Shanguo Zhang, Tianyi Jiang, Depeng Yang, Liangyu Cao, Ming Li, Jiachao Tang, Qi Gu, Aitong Xu, Yu Li, Hongyuan Jiang

**Affiliations:** a School of Mechatronics Engineering, Harbin Institute of Technology, No.92 West Da-zhi Street, Harbin, 150001, People's Republic of China; b School of Life Sciences, Harbin Institute of Technology, No.2 Yikuang Street, Harbin, 150001, People's Republic of China

**Keywords:** Engineered exosome, Drug delivery, Triboelectric nanogenerator, Microneedles

## Abstract

Bacterial infection and persistent inflammation severely hinder tissue regeneration; therefore, a synergistic therapeutic strategy that combines antibacterial and pro-healing functions is crucial for the effective treatment of infected wounds. In this study, we construct a self-powered microneedle drug delivery platform based on a Dual-domain engineered exosome (D^2^-Exo) that integrates antimicrobial and healing-promoting capabilities. The exosome is internally loaded with asiaticoside and surface-modified with an antimicrobial peptide. The self-powered microneedle delivery platform, driven by a triboelectric nanogenerator, enables precise and efficient release of the D^2^-Exo. *In vitro* and *in vivo* experiments show that electrical stimulation significantly enhances exosome release and cellular uptake. The D^2^-Exo-loaded platform effectively reduces inflammation, inhibits bacterial infection, and promotes granulation tissue formation and collagen deposition, thereby accelerating the healing of infected wounds. This study presents a promising strategy for the intelligent treatment of infected wounds.

## Highlights

• Dual-domain exosomes are engineered with antibacterial and healing-promoting properties.
• A self-powered microneedle delivery platform enables controlled release of Dual-domain exosomes.
• The platform effectively eliminates bacteria, suppresses inflammation, and enhances cellular activity.
• This strategy significantly promotes infected wound healing.

## Graphical abstract

We developed a dual-domain-exosome-based self-powered microneedle delivery platform for the treatment of infected wounds with dual functionalities by loading asiaticoside internally and grafting antimicrobial peptides on its surface for synergistic antibacterial and pro-healing effects. Delivered via a self-powered microneedle platform, the Dual-domain exosome enables electrically enhanced release and cellular uptake, effectively reducing infection, suppressing inflammation, and accelerating wound regeneration.

**Figure:**
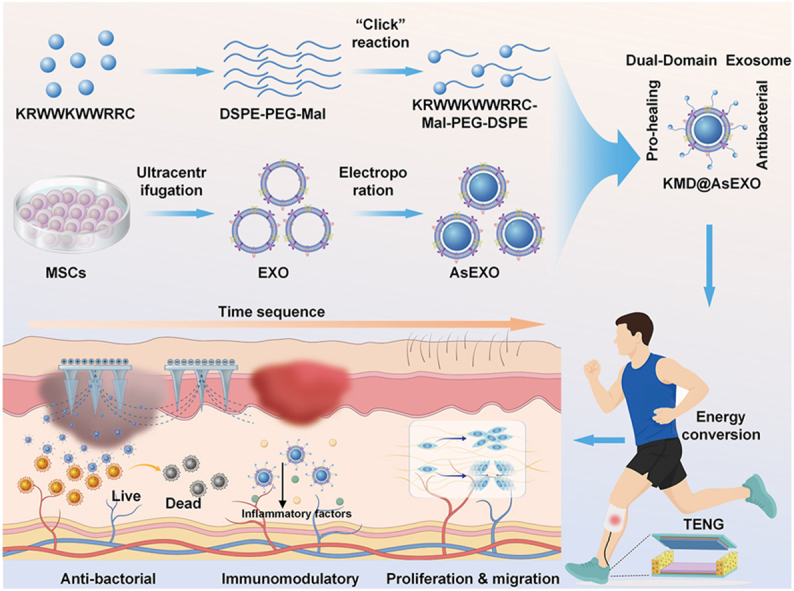

## 1 Introduction

Infected wounds are a common but challenging clinical condition, particularly in cases such as chronic diabetic ulcers, burns, and postoperative infections [[1], [2], [3], [4]]. Their healing is hindered by a complex pathological microenvironment involving bacterial colonization, biofilm formation, persistent inflammation, and impaired tissue repair [[5], [6], [7], [8], [9]]. These factors prolong the healing process and increase the risk of severe complications, including sepsis and tissue necrosis. Conventional treatments rely on antibiotics to control infection and pro-regenerative agents to promote healing [[10], [11], [12], [13]]. However, these approaches often exhibit short-lasting effects, limited tissue penetration, and high drug resistance, making it challenging to achieve effective infection control and tissue repair simultaneously.

Cutaneous injury generates a transepithelial potential (endogenous electric field). Low-intensity exogenous electrical stimulation augments this field, directing the galvanotactic migration of keratinocytes, fibroblasts, and endothelial cells and promoting proliferation, angiogenesis, and collagen deposition, thereby accelerating wound closure [14,15]. Thus, integrating electrical stimulation into wound-care regimens is mechanistically justified and clinically warranted. However, conventional power supplies for electrical stimulation are bulky and tethered, and their resistive heating during operation may cause secondary wound injury [16]. In contrast, triboelectric nanogenerators (TENG) are compact and wearable and deliver high-voltage, low-current outputs, thereby addressing the foregoing limitations. The TENG converts biomechanical motion into electricity via contact electrification and electrostatic induction [[17], [18], [19]]. Its main modes are contact–separation, lateral sliding, single-electrode, and freestanding triboelectric-layer operation. Repeated contact between dissimilar triboelectric layers creates opposite surface charges. Upon separation, the device generates an alternating electric output with high internal impedance. In biomedical use, this electric output is rectified and current-limited, then delivered to tissues or microdevices for enhanced transdermal transport [20,21]. Recent studies using microneedle platforms show that TENGs enable control of drug delivery. Zhang et al. fabricated electrically responsive microneedles that exploit the tunable electrostatic adsorption of polypyrrole under an applied electric field from a TENG to trigger the release of engineered extracellular vesicles [22]. Likewise, Gan et al. [23] and Liu et al. [24] reported that microneedles powered by a TENG can accelerate drug release by inducing electroosmotic flow in interstitial fluid under the TENG-generated electric field. Thus, the integration of microneedles with TENGs allows for the active regulation of drug release through the electric output from mechanical motion [[25], [26], [27], [28]]. However, the electric field from TENG alone affords limited efficacy against infected wounds; therefore, combination with antibacterial and pro-healing modalities is needed [16].

Among emerging approaches, exosome-based therapies have gained considerable attention due to their natural phospholipid bilayer structure, low immunogenicity, and efficient transmembrane delivery [[29], [30], [31], [32]]. Exosomes derived from mesenchymal stem cells (MSCs) exhibit intrinsic regenerative properties, including modulation of inflammation, promotion of angiogenesis, and enhancement of collagen deposition [33,34]. To enhance the antimicrobial and pro-healing efficacy of exosomes, rational engineering is essential. Antimicrobial peptides offer broad-spectrum activity, rapid bacterial clearance, and low resistance, making them promising alternatives to conventional antibiotics [[35], [36], [37]]. Besides, asiaticoside, a plant-derived compound, can promote fibroblast proliferation and collagen synthesis, supporting tissue regeneration and inflammation reduction [[38], [39], [40]]. Incorporating both antimicrobial peptides and asiaticoside can significantly improve the therapeutic potential of exosomes. As shown in Table S1, despite the growing interest in exosome-based therapies for wound healing, current strategies still exhibit several significant limitations. Methods for endowing exosomes with antibacterial properties mainly rely on metal-based antibacterials (e.g., AgNPs), which raise concerns regarding cytotoxicity, resistance, and compatibility. Meanwhile, exosome designs rarely combine antibacterial and reparative functions, and conventional hydrogels lack the penetration and adaptability required for chronic wounds.

In this study, we designed a Dual-domain engineered exosome (D^2^-Exo) exosome with antimicrobial and pro-healing properties and developed a self-powered microneedle delivery platform to enable efficient and controllable release. First, asiaticoside was efficiently encapsulated into MSC-derived exosomes via electroporation. An antimicrobial peptide complex, synthesized through a maleimide–thiol click reaction, was then grafted onto the exosome surface. Subsequently, the D^2^-Exo was loaded into a self-powered microneedle delivery platform to achieve active drug release via TENG-induced electrical stimulation. It was found that this strategy could accelerate wound healing by effectively killing bacteria, modulating inflammatory factors, and promoting cell proliferation, migration, and collagen deposition. This study is expected to provide a new method for the efficient treatment of infected wounds.

## 2 Results and discussion

### 2.1 Synthesis and characterization of D^2^-Exo

**Fig. 1:**
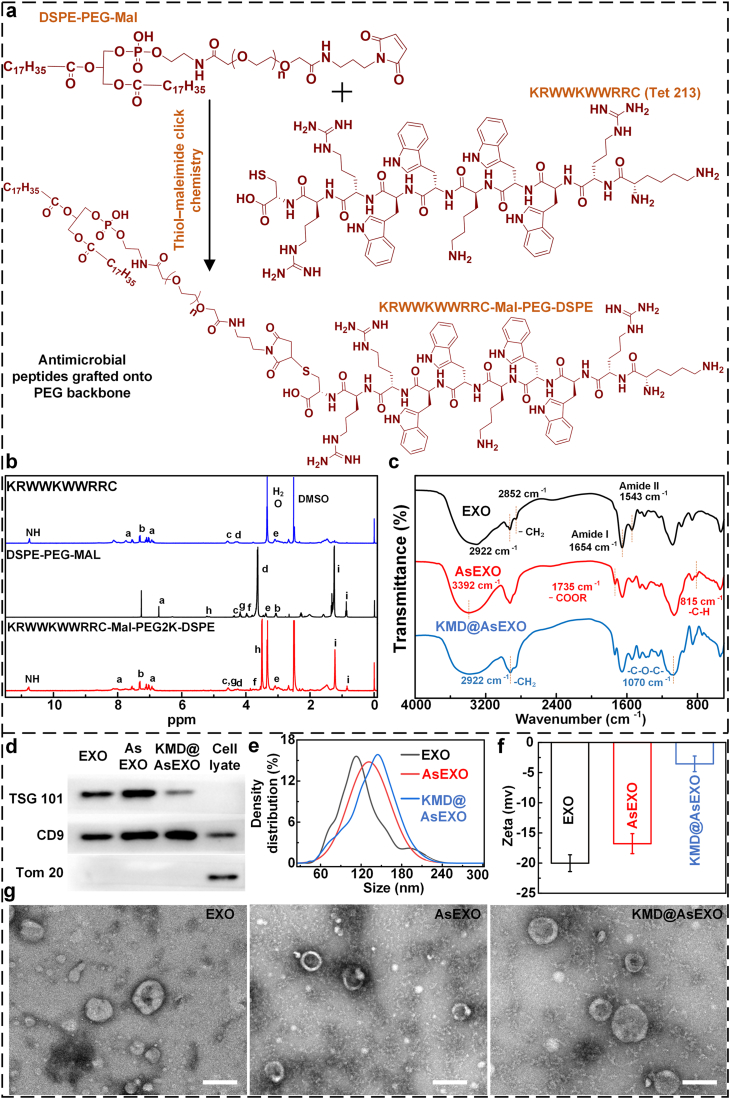
Construction and characterization of KMD@AsEXO. (a) Schematic illustration of the coupling reaction between the antimicrobial peptide and DSPE-PEG-Mal; (b) 1H NMR spectrum confirming successful conjugation; (c) FTIR spectrum showing characteristic functional groups; (d) Western blot analysis of exosomal marker proteins; (e) Dynamic light scattering (DLS) analysis of particle size distribution; (f) Zeta potential measurements; (g) Transmission electron microscopy (TEM) image of different exosomes samples, scale bar: 100 nm.

TET213 (amino acid sequence: KRWWKWWRRC) is a synthetic short-chain cationic antimicrobial peptide with broad-spectrum activity. It shows strong efficacy against gram-positive, gram-negative, and drug-resistant bacteria [41,42]. Based on these properties, it was selected for grafting onto the exosome surface. To graft the antimicrobial peptide onto the exosome surface, it was first conjugated to a lipid-insertable material capable of integrating into the exosomal phospholipid bilayer. As shown in Fig. 1a, the amphiphilic material DSPE-PEG-Mal was covalently linked to the antimicrobial peptide TET213, which contains a terminal sulfhydryl group, via a thiol–maleimide click reaction to form KRWWKWWRRC-PEG-DSPE. DSPE anchors into the exosomal phospholipid bilayer, while the PEG backbone provides flexible spacing to ensure full exposure of the peptide on the exosome surface. As illustrated in Fig. 1b, the successful synthesis of KRWWKWWRRC-Mal-PEG_2_K-DSPE was confirmed by comparative analysis of the 1H NMR spectra of the raw materials (DSPE-PEG-Mal and KRWWKWWRRC) and the final product. In the spectrum of KRWWKWWRRC-Mal-PEG_2_K-DSPE, a characteristic signal corresponding to the active hydrogen (NH) in the peptide backbone of KRWWKWWRRC appeared at 10.77 ppm. The aromatic protons of tryptophan residues and olefinic protons were observed in the range of 8.2–6.8 ppm. Signals at 4.56 ppm and 4.26 ppm corresponded to the methylene protons adjacent to nitrogen atoms (–CH_2_–NH–) within the peptide structure.

The characteristic methylene protons of the PEG backbone (–CH_2_–CH_2_–O–) from DSPE-PEG-Mal appeared at 3.50 ppm, while the methylene and methyl protons from the lipid chains were observed at 1.22 ppm and 0.85 ppm, respectively. These findings indicate that the 1H NMR spectrum of the product retained the signature peaks of both DSPE-PEG-Mal and KRWWKWWRRC. Additionally, the disappearance of the characteristic alkene proton peak of the maleimide ring at 6.72 ppm in the DSPE-PEG-Mal spectrum, along with a slight shift in the aromatic region, further supports the successful conjugation between the peptide and DSPE-PEG-Mal, resulting in the formation of KRWWKWWRRC-Mal-PEG_2_K-DSPE. Then, asiaticoside can be loaded into exosomes by electroporation to construct asiaticoside-loaded exosome (AsEXO), while KRWWKWWRRC-Mal-PEG2K-DSPE can be co-incubated with AsEXO to realize membrane surface modification, and ultimately obtain the D^2^-Exo (KMD@AsEXO) with both antimicrobial and pro-healing functions.

To confirm the physicochemical properties of KMD@AsEXO, we performed a series of characterizations. As shown in Fig. 1c, Fourier transform infrared (FTIR) spectroscopy showed that pure exosomes (EXO) exhibited characteristic asymmetric and symmetric stretching vibration peaks of lipid –CH_2_ at ∼2922 and ∼2852 cm^−1^, respectively. Peaks at 1654 cm^−1^ (amide I) and 1543 cm^−1^ (amide II) corresponded to the main chain of membrane proteins. In the AsEXO group, the broad hydrogen-bond-related peaks at 3292 cm^−1^ became more pronounced, the ester carbonyl peak at 1735 cm^−1^ intensified, and a sugar ring out-of-plane vibration peak appeared at ∼815 cm^−1^, confirming the successful incorporation of asiaticoside into the exosomes. In the KMD@AsEXO group, further grafting of KRWWKWWRRC-PEG-DSPE onto the exosome membrane led to an increased amide I/II peak area, the appearance of a PEG ether bond peak at ∼1070 cm^−1^, and an enhanced –CH_2_ stretching peak at ∼2922 cm^−1^, confirming the successful insertion of the DSPE hydrophobic anchor into the lipid bilayer. Furthermore, FTIR analysis of a solid-state physical mixture of KRWWKWWRRC–PEG–DSPE, asiaticoside, and exosomes showed no additional functional-group absorption bands relative to KMD@AsEXO; only minor wavenumber shifts attributable to microenvironmental effects were observed (Fig. S1). Additionally, confocal microscopy reveals the colocalization of FITC-labeled KRWWKWWRRC-PEG-DSPE with PKH26-labeled exosomes (Note S1 and Fig. S2). These results indicate that no covalent bonds are formed in KMD@AsEXO, and AsEXO was modified by KRWWKWWRRC-PEG-DSPE via lipid insertion, which is consistent with the previous reports [43,44]. Western blot analysis (Fig. 1d) showed strong expression of classical exosomal markers TSG101 and CD9 across all three groups. The mitochondrial protein Tom 20 was absent in exosome samples and detected only in the cell lysate, indicating high preparation purity. These results confirm that drug loading and surface modification did not alter the characteristic protein profile of the exosomes. The average particle size increased slightly from ∼112 nm for EXO to ∼145 nm for KMD@AsEXO, indicating that drug loading and peptide modification had minimal impact on exosome size (Fig. 1e). Besides, exosomes carried a strong negative charge (∼−16.5 mV). After grafting with the positively charged peptide and loading with asiaticoside, the surface potential increased to approximately −5 mV (Fig. 1f). Transmission electron microscopy confirmed that all three exosome groups retained a typical vesicular structure with well-defined boundaries and good dispersion (Fig. 1g), indicating that the treatment process did not induce significant aggregation or structural damage.

### 2.2 Design of the platform

**Fig. 2:**
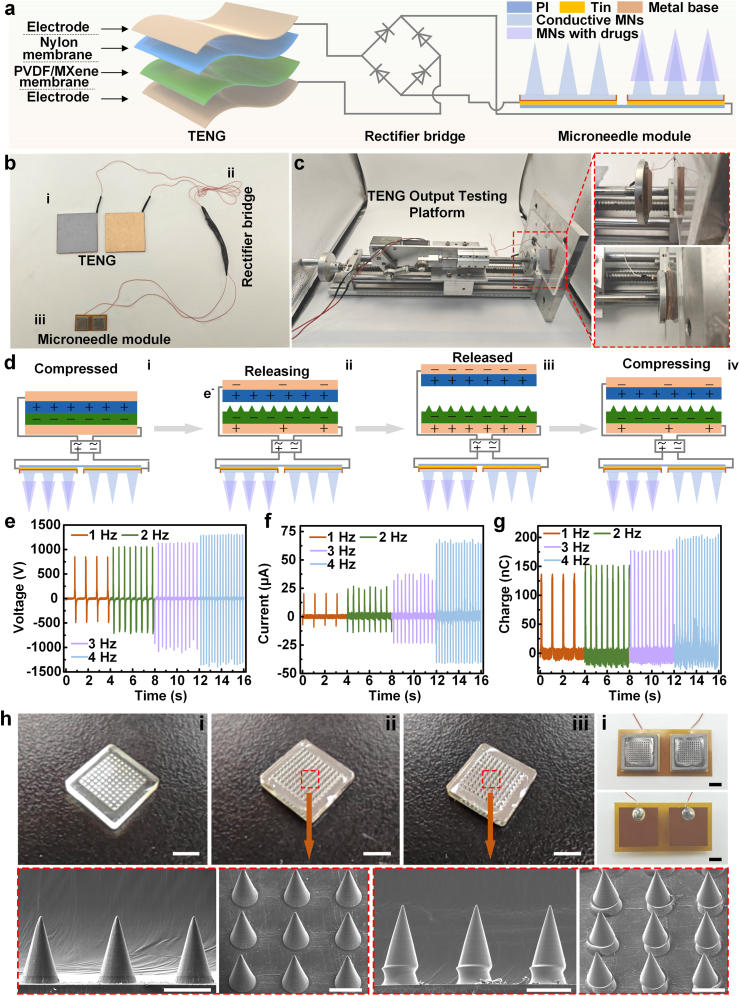
Construction and performance characterization of the self-powered microneedle delivery platform. (a) Schematic illustration of the platform composition; (b) Photograph of the assembled platform; (c) Experimental setup for evaluating the output performance of the TENG; (d) Schematic diagram of charge transfer mechanism of the TENG; (e–g) Output voltage, current, and charge at different operating frequencies; (h) Macroscopic and scanning SEM images of various microneedle arrays (scale bars: top row, 4 mm; bottom row, 500 μm); (i) Assembled top and bottom view of the microneedle module (scale bar: 4 mm).

To achieve efficient and controlled release of KMD@AsEXO, a self-powered microneedle delivery platform based on TENG was developed in this study. As illustrated in Fig. 2a and b, the platform comprises three main components: (i) a contact-separation mode TENG, featuring a PVDF/MXene composite film and a nylon film as dielectric layers, assembled in a typical vertical contact structure; (ii) a bridge rectifier module that converts the alternating current of the TENG into pulsed direct current (Fig. S3); and (iii) a drug delivery module incorporating conductive microneedles, which is connected to the rectifier output to enable electrical-controlled drug release. The performance of the TENG and the microneedle module is detailed below. As shown in Fig. 2c, the TENG output was evaluated using a custom-built test platform. Specifically, the TENG is powered by human biomechanical motion (hand tapping or footfalls during walking) in practical applications [45]. For study uniformity, consistent test parameters, and experimental reproducibility, we therefore used a motor-driven crank–slider mechanism to provide a stable, controlled mechanical input as reported before [46,47]. The surface of the PVDF/MXene composite film exhibits nanoscale protrusions, which effectively enhance the electrical output of the TENG (Fig. S4). As illustrated in Fig. 2d, the electrical output is generated through the synergistic interaction of triboelectric charging and electrostatic induction. Specifically, copper tape serves as the electrode for both the PVDF/MXene composite film and the nylon film. When connected to the microneedle module via the bridge rectifier, the TENG operates in contact-separation mode, utilizing the interaction between the PVDF/MXene and nylon films to generate electrical output. Owing to the high triboelectric negativity of the PVDF/MXene composite film, its contact with the nylon film induces surface charge transfer, wherein the PVDF/MXene film gains electrons while the nylon surface loses electrons. This contact generates negative and positive charges on the respective surfaces of the dielectric layers (Fig. 2d i). Upon separation, electrostatic induction induces a potential difference, which drives charge flow through the external circuit, resulting in a transient current (Fig. 2d ii). When the layers are fully separated, the induced charges in the conductive electrode reach equilibrium with the surface charges on the dielectric materials (Fig. 2d iii). As the layers reapproach, the accumulated charges in the conductive layer are discharged through the external circuit to neutralize the potential difference (Fig. 2d iv). In our experiments, the output performance of the TENG was influenced by the contact frequency. As the frequency increased from 1 Hz to 4 Hz, significant enhancements were observed across multiple output parameters: the voltage rose from 840 V to 1290 V (Fig. 2e), the current increased from 20 μA to 64 μA (Fig. 2f), and the transferred charge grew from approximately 135 nC–205 nC (Fig. 2g). This enhancement can be attributed to the increased momentum at higher contact frequencies, which induces greater deformation of the friction layers [16]. Such deformation may enlarge the effective contact area between the triboelectric materials, thereby enhancing the electrical output. Besides, the current output decreases as environmental humidity increases. When humidity reaches 100 %, the current output drops to around 9 μA. Therefore, to ensure the efficient operation of TENG, it should be used in a dry environment (Fig. S5). The microneedle module primarily consists of conductive single-layer microneedles, conductive double-layer microneedles, and flexible circuit boards. Fig. 2h illustrates the macroscopic morphology and microscopic structural characteristics of the two types of microneedles. The initial single-layer microneedles were fabricated using a mold-based method with PEGDA hydrogels as the primary materials (Fig. 2h i). The initially prepared microneedles were transparent and measured approximately 800 μm in length. To impart electrical conductivity, a platinum layer was deposited onto their surface via magnetron sputtering (Fig. 2h ii). Subsequently, prepolymer tips loaded with KMD@AsEXO were assembled onto the conductive single-layer microneedles using a repeated mold-based method, resulting in the formation of double-layer conductive microneedles (Fig. 2h iii). Notably, guided by the balance of the conductivity and strength properties, the formulation was optimized to GelMA 16 % (v/v) and PEGDA 12 % (v/v), and the corresponding optimized internal resistance of the microneedle is 52.7 kΩ (Note S2). The microneedle tip remained stable in PBS (37 °C) for at least 7 days without detectable degradation, ensuring the controlled release of the drug (Fig. S6). In practical applications, the conductive double-layer microneedles loaded with KMD@AsEXO were positioned on the positive electrode of the flexible circuit board using a custom-designed metal mold (Fig. 2i and Fig. S7), while the single-layer conductive microneedles were aligned with the negative electrode. In this configuration, the direct current output from the rectifier bridge was transmitted through the flexible circuit board to the microneedles, thereby facilitating electrically controlled drug release. The single-layer and double-layer conductive microneedles sustained axial compressive forces of about 0.25 N and 0.21 N per needle, respectively (Fig. S8). These values are well above the 0.045 N threshold reported for cuticle penetration [48,49]. The flexible circuit board transmits electrical signals from the top layer to the bottom layer via through-holes (Fig. S9). Notably, although exosomes typically possess a negative surface charge, modification with positively charged peptides renders the KMD@AsEXO nearly electrically neutral. Generally, a zeta potential between −10 mV and 10 mV is considered electrically neutral [50]. Thus, the D^2^-Exo experience minimal electrophoretic force and should be positioned on the positive electrode to facilitate their release primarily via electroosmotic flow. The corresponding validation experiments will be discussed in a subsequent section.

### 2.3 KMD@AsEXO release from the platform

**Fig. 3:**
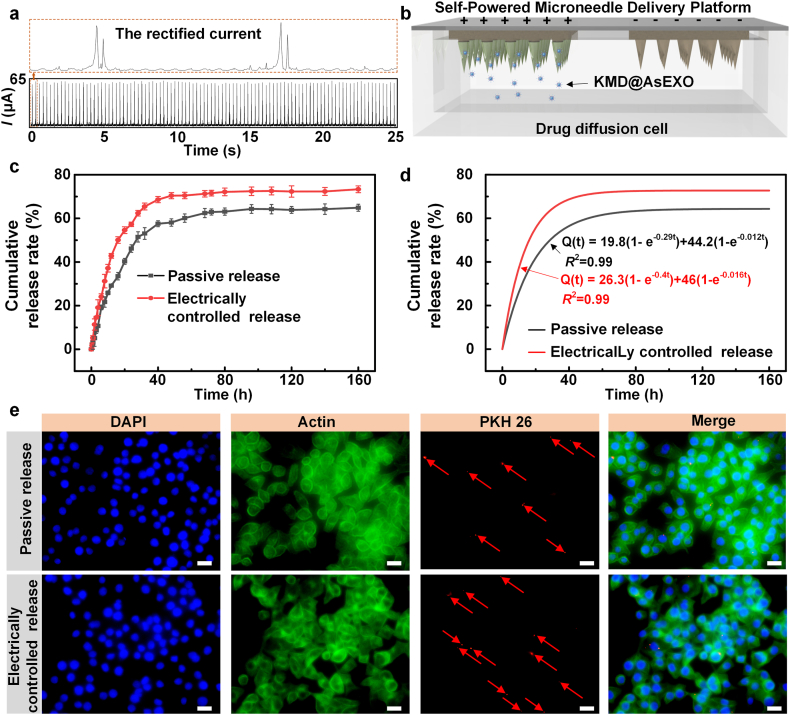
Characterization of the KMD@AsEXO release performance from the self-powered microneedle system. (a) Electrical output signal of the TENG after rectification via a bridge circuit; (b) Schematic illustration of the drug release experimental setup; (c) Cumulative drug release profiles under passive diffusion and electrically controlled conditions; (n = 3 independent samples, means ± SD) (d) Kinetic fitting of the release data using a biphasic release mode; (e) Cellular uptake of KMD@AsEXO under different release conditions, as observed by fluorescence microscopy (scale bar: 20 μm).

The release behavior of KMD@AsEXO within the self-powered microneedle delivery platform plays a crucial role in the treatment of infectious wounds. In this study, the 4 Hz electrical output from the TENG was converted into a stable pulsed direct current signal via bridge rectification (Fig. 3a), resulting in a peak current of approximately 65 μA with a pulse interval of 250 ms, closely resembling the frequency of human light tapping or gait. This output was adopted for subsequent experiments. As shown in Fig. 3b, to simulate drug release under physiological conditions, the drug-loaded microneedle module was mounted on top of a 3D-printed release chamber, with the needle tips immersed in PBS buffer at 37 °C. Electrical output was delivered to the microneedle patch via the flexible circuit board. After 4 h of release, the cumulative release rate in the passive group was approximately 10.7 %, whereas the electrically controlled group achieved a significantly higher release rate of 19.1 %, as shown in Fig. 3c. Throughout the experiment, the release enhancement effect of electrical stimulation persisted and gradually approached equilibrium at approximately 108 h. By this time, the cumulative release rate reached 64.3 % in the electrically controlled group, compared to 72.4 % in the passive release group. To quantify the release kinetics, the release profile was fitted using a biphasic drug release model, characterized by an initial burst phase followed by a sustained release phase (Fig. 3d). The mathematical formulation of the model is:

$$Q\left(t\right)=A_{1}\left(1-e^{-k_{1}t}\right)+A_{2}\left(1-e^{-k_{2}t}\right)$$

where Q(t) is the cumulative release rate (%) at a given time t, $A_{1}$, $k_{1}$ are the total amount of release and the release rate of the fast phase, and $A_{2}$, $k_{2}$ are the total amount of release and the release rate of the slow phase [51].

The fitting parameters in the electrically controlled group were higher than those in the passive group, and the fast-phase rate constant *k*_1_ increased from 0.29 to 0.40, indicating that the electric field effectively accelerated drug release. Both groups achieved high goodness-of-fit, with R^2^ values of 0.99, confirming the model’s suitability. Notably, when the nearly neutral-charged KMD@AsEXO was placed on the negative electrode instead of the positive electrode, the release-enhancing effect was markedly reduced (Fig. S10). As shown in Fig. S11, the on-demand drug release capability of the designed platform can be demonstrated by intermittently triggering the TENG and observing the drug release pattern. Passive release produced only slow increases in cumulative release. When the TENG was switched on, the slope rose markedly. The reproducible alternation between low and high release rates demonstrates electrically gated control over drug release. Accordingly, within the self-powered microneedle platform, the microneedles spatially target the wound site, while the TENG provides time control over drug release, thereby enabling the spatiotemporally controlled release of D^2^-Exo. Furthermore, to demonstrate the biological relevance of enhanced drug release, we conducted cellular uptake experiments. As shown in Fig. 3e, human umbilical vein endothelial cells (HUVECs) were incubated for 24 h in a release solution containing PKH26-labeled KMD@AsEXO, followed by fluorescence imaging. In the passive release group, only weak red fluorescence was observed, whereas the electrical-controlled group exhibited a markedly stronger fluorescence signal. This indicates that electrical stimulation significantly increased exosome release, thereby enhancing cellular uptake efficiency.

### 2.4 Antibacterial activity

**Fig. 4:**
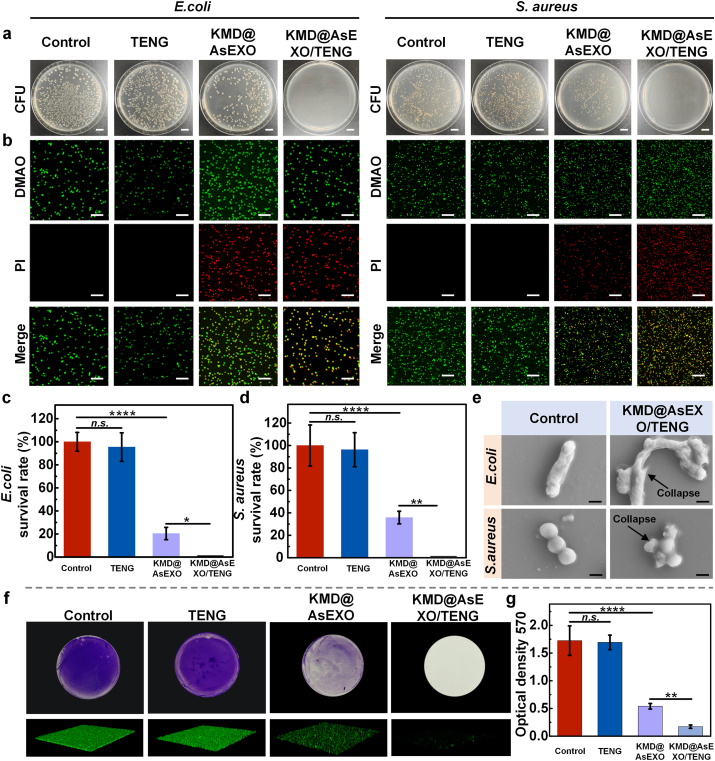
Antibacterial effects of the self-powered microneedle delivery platform loaded with KMD@AsEXO. (a) Bacterial colony count after treatment; scale bar: 10 mm. (b) Live/dead bacterial staining using DMAO/PI, visualized by fluorescence microscopy; scale bar: 20 μm. (c, d) Quantitative analysis of bacterial survival rates for *E. coli* and *S. aureus*, respectively. (e) SEM images showing morphological changes in bacteria after treatment; scale bar: 50 μm. (f) Biofilm staining and fluorescence imaging to assess biofilm formation. (g) Quantification of biofilm biomass based on optical density measurements. (n = 3 independent samples, means ± SD, ∗p < 0.05, ∗∗p < 0.01, ∗∗∗∗p < 0.0001).

To evaluate the antibacterial efficacy of the KMD@AsEXO-loaded self-powered microneedle delivery platform, two representative bacterial strains—*Escherichia coli* (*E. coli*) and *Staphylococcus aureus* (*S. aureus*)—were selected as model organisms. The bacterial suspension was placed in a custom 3D-printed mold (identical to that used for drug release experiments, Fig. 3b), and the microneedle module of the self-powered drug delivery system was positioned on top of the 3D-printed mold, with the needle tips immersed in the bacterial solution. As shown in Fig. 4a, the antibacterial efficacy was evaluated using the colony-forming unit (CFU) counting method. The control group and the TENG group exhibited a high number of bacterial colonies indicating that the external electric field alone had a minimal bactericidal effect. KMD@AsEXO group resulted in partial inhibition of bacterial growth, though residual colonies remained. In contrast, the KMD@AsEXO/TENG group achieved complete bacterial clearance. DMAO is a green fluorescent dye that can stain both live and dead bacteria, whereas propidium iodide (PI) is membrane-impermeable and selectively stains dead bacteria, emitting red fluorescence. The combination of DMAO and PI enables the differentiation between live and dead bacteria. As shown in Fig. 4b, the Control and TENG groups exhibited minimal red fluorescence, indicating low bacterial death. In the KMD@AsEXO group, a portion of the green fluorescence shifted to red, reflecting partial bacterial killing. Notably, in the KMD@AsEXO/TENG group, nearly all green fluorescence signals converted to red, suggesting extensive bacterial death. Quantitative analysis of the CFU results is presented in Fig. 4c and d. Treatment with TENG did not significantly affect the survival of *E. coli* or *S. aureus*. Following KMD@AsEXO treatment, the survival rate of *E. coli* was approximately 20 %, while that of *S. aureus* was around 35 %. In contrast, no bacterial colonies were detected in the KMD@AsEXO/TENG group, indicating complete bacterial eradication. These results indicate that although the electrical signal alone exhibits limited bactericidal activity, TENG-induced stimulation markedly enhances KMD@AsEXO release, thereby improving antibacterial efficacy. It is worth noting that KMD@AsEXO exhibited higher antibacterial efficacy against *E. coli* compared to *S. aureus*. This difference may be attributed to the mechanism of action of the antibacterial peptide, which primarily disrupts bacterial membranes [52,53]. The thin outer membrane of *E. coli* makes it highly vulnerable to peptide-induced disruption, whereas the peptidoglycan wall of *S. aureus* confers greater resistance [54]. To directly observe morphological changes in bacteria following treatment, SEM was employed. As shown in Fig. 4e, untreated bacteria exhibited an intact, plump morphology with well-defined boundaries. In contrast, bacteria in the KMD@AsEXO/TENG group displayed pronounced collapse, surface damage, and structural distortion, indicating significant disruption of the cell membrane. Additionally, to assess the inhibitory effect of the platform on bacterial biofilms, crystal violet staining was performed to evaluate biofilm formation across the four groups (Fig. 4f). The results showed that only the KMD@AsEXO/TENG group exhibited minimal staining residue, whereas the other groups displayed evident purple deposition, indicating the presence of biofilms. Quantitative optical density measurements (Fig. 4g) further confirmed this trend, demonstrating that the KMD@AsEXO-loaded self-powered microneedle delivery platform not only effectively eradicates planktonic bacteria but also significantly inhibits bacterial biofilm formation.

### 2.5 Biocompatibility and biological properties

**Fig. 5:**
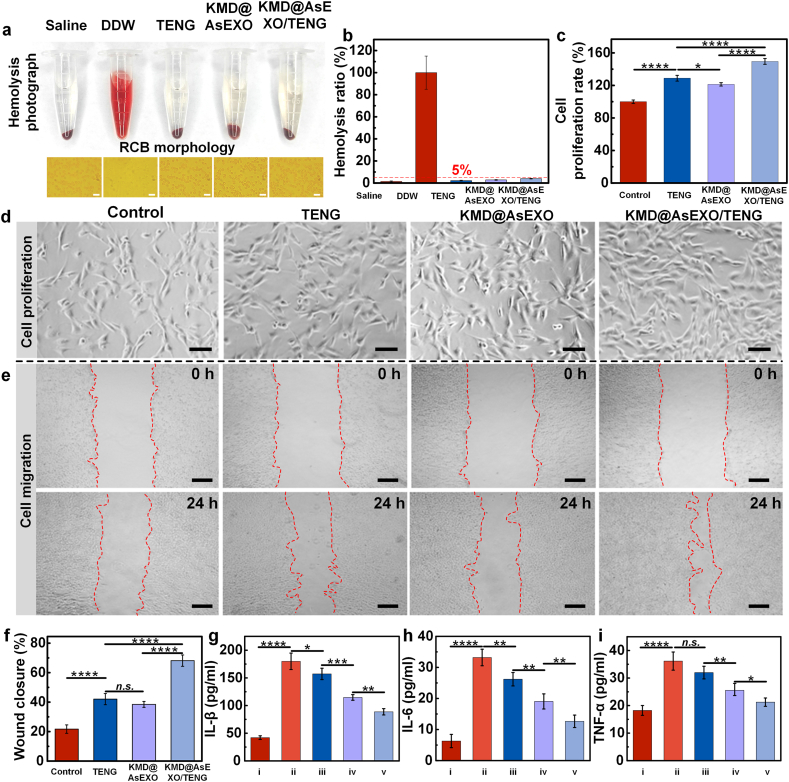
Evaluation of *in vitro* biocompatibility and pro-healing effects of the self-powered microneedle delivery system. (a) Macroscopic and microscopic images of red blood cells after various treatments in physiological saline (scale bar: 10 μm); (b) Quantitative analysis of hemolysis rates; (c) NIH-3T3 cell proliferation and corresponding cell morphology (d, scale bar: 100 μm); (e) Microscopic images of wound closure in the scratch assay (scale bar: 500 μm); (f) Quantitative analysis of cell migration rates; (g–i) Expression levels of inflammatory cytokines IL-6, IL-1β, and TNF-α under LPS stimulation, assessed by ELISA. Groups i–v correspond to the Control, LPS, TENG, KMD@AsEXO, and KMD@AsEXO/TENG groups, respectively. (n = 3 biologically independent samples, means ± SD, ∗p < 0.05, ∗∗p < 0.01, ∗∗∗p < 0.001, ∗∗∗∗p < 0.0001). (For interpretation of the references to colour in this figure legend, the reader is referred to the Web version of this article.)

To assess the biocompatibility and pro-healing potential of the KMD@AsEXO-loaded self-powered microneedle delivery platform, a series of *in vitro* experiments were conducted. Hemolysis assays were performed to evaluate the blood compatibility of the materials. As shown in Fig. 5a and b, the hemolysis rate in all experimental groups remained below 5 %, indicating that TENG-derived electrical stimulation, KMD@AsEXO, and their combined application exhibit excellent blood compatibility. As shown in Note S3, KMD@EXO exhibited synergistic therapy with potent antibacterial activity and cell proliferation, underscoring its biological significance. Additionally, when NIH3T3 cells were treated with the three experimental conditions, no cytotoxic effects were observed. Instead, all treatments promoted cell proliferation compared to the control group. Specifically, the TENG group exhibited a proliferation rate approximately 10 % higher than that of the KMD@AsEXO group, while the KMD@AsEXO/TENG group showed an increase of approximately 50 % relative to the control, as shown in Fig. 5c. These findings indicate that both TENG-derived electrical stimulation and KMD@AsEXO independently promote cell proliferation, while their combined application further enhances this effect. It is worth noting that the drug loading on the surface of exosomes does not affect their intrinsic biological activity (Note S4). Bright-field imaging (Fig. 5d) revealed that NIH3T3 cells across all treatment groups maintained normal spindle-shaped morphology, with no signs of apoptosis or cellular fragmentation, suggesting that the proposed strategy does not exert adverse effects on cell morphology. Therefore, the self-powered microneedle delivery platform loaded with KMD@AsEXO demonstrates excellent biocompatibility. To further evaluate its wound healing potential *in vitro*, a scratch assay was performed. As shown in Fig. 5e, the migration of NIH3T3 cells varied across treatment groups, reflecting the differential effects of each intervention on cell motility and wound closure. At the initial time point (0 h), the scratch width was comparable across all groups. However, after 24 h of treatment, the wound closure rate of approximately 68 % in the KMD@AsEXO/TENG group, significantly higher than that of the KMD@AsEXO group (∼38 %) and the TENG group (∼42 %), as shown in Fig. 5f. These results indicate that electrical stimulation not only facilitates the release of KMD@AsEXO but also directly promotes cell migration and tissue repair. To further evaluate the anti-inflammatory effects of the platform, a macrophage inflammation model was established using LPS stimulation, and the expression levels of key pro-inflammatory cytokines (IL-1β, IL-6, and TNF-α) were assessed. As shown in Fig. 5g–i, LPS treatment significantly upregulated the expression of all three cytokines. However, both the TENG and KMD@AsEXO groups effectively downregulated their expression, indicating that each treatment possesses notable anti-inflammatory potential. Notably, the expression levels of inflammatory cytokines were further reduced in the KMD@AsEXO/TENG group, highlighting a synergistic anti-inflammatory effect. Given that KMD@AsEXO comprises multiple components, including exosome, asiaticoside, and KRWWKWWRRC-PEG-DSPE. It is essential to delineate the specific contribution of each component to the observed effects on cell proliferation, migration, and inflammation suppression. As shown in Fig. S12 and S13, KRWWKWWRRC-PEG-DSPE alone did not significantly enhance cell activity in proliferation and migration assays, nor did it exhibit notable anti-inflammatory effects. While unmodified exosomes demonstrated some capacity to promote cell proliferation, migration, and reduce inflammation, these effects were relatively modest. In contrast, the observed therapeutic benefits were primarily attributed to asiaticosides, emphasizing the importance of the encapsulation of asiaticosides within exosomes to achieve effective and sustained biological activity.

### 2.6 In vivo evaluation of infected wound healing

**Fig. 6:**
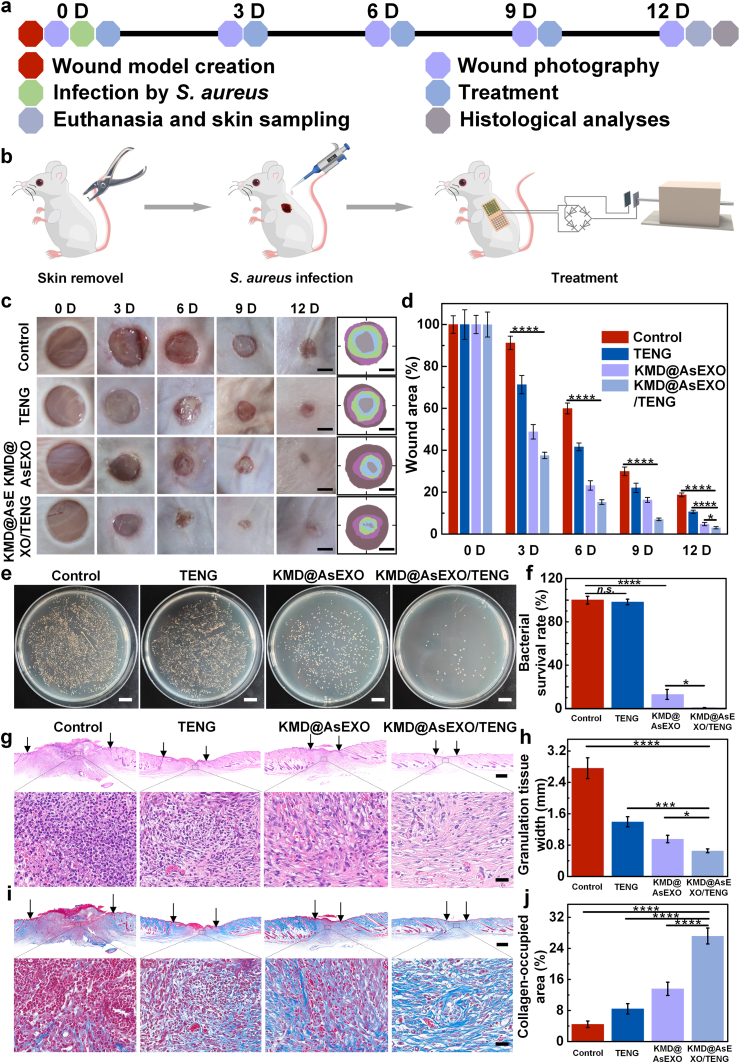
*In vivo* therapeutic evaluation of the self-powered microneedle platform in an infectious wound model. (a) Schematic illustration of the construction and treatment workflow for the infectious wound model; (b) Diagram of mouse infection modeling and corresponding treatment procedures; (c) Representative optical images of wound healing across different treatment groups at designated time points (scale bar: 2 mm); (d) Quantitative analysis of wound area over time; (e) Optical images and (f) corrosponding quantitative data showing bacterial colonies at the wound site on day 6 following various treatment. All scale bars are 10 mm. (g) Hematoxylin and eosin staining to assess tissue structure and inflammatory response (scale bars: top row, 400 μm; bottom row, 20 μm); (h) Quantification of granulation tissue width; (i) Masson’s trichrome staining to evaluate collagen remodeling (scale bars: top row, 400 μm; bottom row, 20 μm); (j) Quantitative analysis of collagen deposition area. (n = 3 biologically independent samples, means ± SD, ∗p < 0.05, ∗∗∗p < 0.001, ∗∗∗∗p < 0.0001).

To evaluate the wound healing effects of the KMD@AsEXO-loaded self-powered microneedle platform, experimental validation was conducted using infectious wound models in mice. As shown in Fig. 6a and b, circular full-thickness skin wounds were created on the backs of mice using a punch on day 0, followed by inoculation with S*. aureus* to establish an infectious wound model. Treatments were administered every three days (Fig. S14a), and tissue samples were collected on day 12 for histological analysis. As shown in Fig. 6c, the initial wound conditions were comparable across all groups. During treatment, the wounds in the Control and TENG groups exhibited slow healing, accompanied by obvious infection and swelling. In contrast, the KMD@AsEXO group demonstrated a moderate wound-healing effect, with visible wound size reduction and alleviation of infection. Notably, the KMD@AsEXO/TENG group showed rapid wound contraction, significant attenuation of the inflammatory response, and near-complete wound closure by day 12. Quantitative analysis (Fig. 6d) further confirmed that the KMD@AsEXO/TENG group exhibited the fastest wound closure rate. Specifically, on day 12, the wound area in the KMD@AsEXO/TENG group had decreased to approximately 3 %, compared with around 5 % in the KMD@AsEXO group and 10 % in the TENG group. Notably, wound healing in the TENG group was accelerated compared to the control group (about 18 %, day 12), indicating that appropriate electrical stimulation promotes wound repair. This phenomenon is attributable to the augmentation by electrical stimulation of the skin’s injury-evoked endogenous electric field [14,15]. Therefore, the accelerated wound healing observed in the KMD@AsEXO/TENG group can be attributed to the synergistic effects of electrical stimulation and enhanced KMD@AsEXO release. To further assess the *in vivo* antibacterial effect, we harvested skin tissue encompassing the entire wound area from each group and prepared tissue homogenates. Serial dilutions in PBS were plated on LB agar and incubated for 24 h. As shown in Fig. 6e and f, TENG did not reduce bacterial counts versus the control group. By contrast, KMD@AsEXO significantly decreased bacterial survival, and the KMD@AsEXO/treatment produced a further reduction. It is worth noting that although combining exosomes with TENG improves healing relative to TENG alone, the absence of intrinsic antibacterial activity and the modest pro-healing potency of native exosomes limit both wound closure and bactericidal outcomes, leaving performance markedly inferior to the KMD@AsEXO and KMD@AsEXO/TENG groups (Fig. S14b-d).

Histological analysis provided insight into the microscopic mechanisms underlying wound repair. Hematoxylin and eosin staining revealed extensive inflammatory cell infiltration, disorganized tissue architecture, and incomplete epidermal formation in the control group, as shown in Fig. 6g. Both the TENG and KMD@AsEXO groups exhibited partial epithelial reconstruction and reduced inflammation compared with the control group. Besides, the KMD@AsEXO group displayed a denser extracellular matrix and fewer infiltrating inflammatory cells, indicating improved tissue regeneration. In contrast, the KMD@AsEXO/TENG group exhibited a fully regenerated and continuous epidermis, a densely organized dermal layer, and minimal inflammatory cell infiltration, indicating that the tissue architecture had recovered to a state most comparable to that of normal skin. Correspondingly, quantitative analysis of granulation tissue width (Fig. 6h) revealed that the control group exhibited the thickest granulation tissue, whereas the KMD@AsEXO/TENG group showed a significantly reduced width. This suggests that the formation of inflammatory hyperplastic tissue was effectively suppressed, thereby facilitating tissue regeneration. Masson’s trichrome staining (Fig. 6i) further supported this observation: the Control and TENG groups displayed sparse and disorganized collagen fibers, while the KMD@AsEXO group demonstrated moderate collagen deposition with relatively aligned fiber orientation. The KMD@AsEXO/TENG group exhibited extensive collagen deposition, as indicated by dense blue staining, with a regular and tightly aligned fiber structure. Quantitative analysis (Fig. 6j) showed that the collagen content in the KMD@AsEXO/TENG group was significantly higher than in the other groups, highlighting its ability to promote collagen remodeling and tissue maturation. Besides, histopathology analysis of typical organs and blood routine examination data proved the safety of KMD@AsEXO/TENG treatment (Fig. S15 and Table S2). Therefore, the self-powered microneedle platform loaded with KMD@AsEXO demonstrated superior therapeutic efficacy.

## 3 Conclusion

In summary, we engineered a D^2^-Exo and delivered it using a self-powered microneedle platform for infected-wound therapy. Electric stimulation from the triboelectric nanogenerator improved exosome release and uptake, raised local drug levels, and accelerated healing. *In vitro* and *in vivo* studies showed reduced infection and inflammation, faster re-epithelialization, and enhanced collagen remodeling. For clinical translation, several issues merit attention. First, a national standard-compliant scale-up with validated potency assays and batch consistency is essential. In addition, the integrated exosome patch must maintain storage stability and sterility. Moreover, human skin compatibility, long-wear comfort, and electrical safety across diverse skin types should be verified. Furthermore, device reliability, adhesion under motion, and ease of use in ambulatory care are critical. Finally, a clearly defined regulatory pathway for a combination biologic–device product is required. Large-animal studies and early human feasibility trials will help address these gaps and guide clinical adoption.

## 4 Materials and methods

### 4.1 Materials

DSPE-PEG2K-Mal and the antimicrobial peptide Tet213 (KRWWKWWRRC) were purchased from Apeptide (Shanghai, China). N,N-Dimethylformamide (DMF, Cat. No. D111999), triethylamine (Cat. No. T103285), and asiaticoside (also referred to as aloe glycoside, Cat. No. A111276) were obtained from Aladdin. The photoinitiator 2-hydroxy-2-methyl-1-phenyl-1-propanone (Darocur 1173, Cat. No. 405655) and polyethylene glycol diacrylate (PEGDA, Cat. No. 455008) were purchased from Sigma-Aldrich. Methacrylated gelatin (GelMA, Order No. WT001) was supplied by Engineering for Life. Mesenchymal stem cell serum-free medium (Cat. No. WSC0008) was obtained from Vistas Biotech Co., Ltd. (Wuhan, China), and Endothelial Cell Medium was purchased from Sciencell. Fetal bovine serum (FBS, Cat. No. 16000044) and high-glucose DMEM (Cat. No. 11965092) were obtained from Thermo Fisher Scientific. FBS was centrifuged at 120,000×*g* for 90 min before use to remove endogenous exosomes. The bacterial viability staining kit (Cat. No. C2030S) and BCA protein quantification kit (Cat. No. P0009) were purchased from Beyotime Biotechnology Co., Ltd. PKH26 membrane dye (Cat. No. D0030) was obtained from Solarbio. Primary antibodies, including anti-Tom20 (Cat. No. ab186735), anti-CD9 (Cat. No. ab223052), and anti-TSG101 (Cat. No. ab133586), were purchased from Abcam. MXene nanosheets were obtained from Foshan Xinxi Technology Co., Ltd., and PVDF powder (Kynar® 761) was provided by Arkema. Nylon membranes were purchased from Dongguan Yixuan Plastic Material Co. ELISA kits for IL-6 (Cat. No. mI098430), TNF-α (Cat. No. ml002095), and IL-1β (Cat. No. ml106733) were obtained from Enzyme-linked Biotechnology Co., Ltd.

### 4.2 Cells, bacteria, and animals

Human umbilical cord mesenchymal stem cells, human umbilical vein endothelial cells, mouse macrophages, and mouse fibroblasts were obtained from the American Type Culture Collection (ATCC, USA). Mouse red blood cells were provided by Hongquan Biotechnology Co., Ltd. (Guangzhou, China). The bacterial strain was purchased from the Microbiological Culture Collection Center (Beijing, China). Ten-week-old male BALB/c mice were supplied by Changsheng Biotechnology (China).

### 4.3 The fabrication of KRWWKWWRRC-PEG-DSPE

To synthesize the peptide-conjugated complex, 50 mg of DSPE-PEG_2_K-Mal was dissolved in 3 mL of DMF, followed by the addition of the antimicrobial peptide (1.1 molar equivalents) and triethylamine (3.0 molar equivalents). The mixture was thoroughly stirred and allowed to react at room temperature for 12 h. Upon completion, the reaction solution was transferred to a dialysis bag (molecular weight cut-off: 1500 Da) and dialyzed against deionized water for 24 h. The dialysate was then collected and lyophilized to obtain the final product.

### 4.4 Cell culture and the preparation of D^2^-Exo

Human umbilical cord mesenchymal stem cells (passages 3–5) were cultured in mesenchymal stem cell serum-free medium at 37 °C with 5 % CO_2_ for 48 h. The supernatant was sequentially centrifuged at 300×*g* for 10 min, 2000×*g* for 20 min, and 10,000×*g* for 30 min to remove cells and debris. Exosomes were subsequently isolated by ultracentrifugation at 120,000×*g* for 90 min, resuspended in PBS, and subjected to a second round of ultracentrifugation under the same conditions to obtain highly purified exosomes (EXO). For drug loading, 200 μg of exosomes (based on total protein content) were mixed with 1 mg of asiaticoside and placed in an electroporation cuvette. Electroporation was performed at 400 V with a pulse duration of 100 ms, for a total of two pulses. The mixture was then incubated at 37 °C for 30 min to facilitate membrane recovery. Unencapsulated asiaticoside was removed using 100 kDa ultrafiltration, yielding asiaticoside-loaded exosomes (AsEXO). KRWWKWWRRC-PEG-DSPE was dissolved in PBS and ultrasonicated at 65 °C for 5 min to form a 1 mg/mL micellar solution. Surface modification of AsEXO was achieved via lipid insertion by incubating with KRWWKWWRRC-PEG-DSPE at a protein-to-lipid mass ratio of 1:3, under gentle shaking at 37 °C for 1 h. Free micelles were removed by size exclusion chromatography, resulting in the final D^2^-Exo, designated KMD@AsEXO.

### 4.5 Preparation of TENG

The dielectric layers of the TENGs used in this study were primarily composed of PVDF/MXene composite films and nylon films. The PVDF/MXene composite films were fabricated using the combined crystallization and diffusion method previously developed and reported by our group [45]. The bottom casting solution consisted of MXene loaded with silver nanoparticles, PVDF, and cellulose acetate dissolved in DMSO. The top casting solution contained MXene and PVDF in DMSO. Both solutions were homogenized by stirring in an 80 °C water bath.0.3 mL of the bottom solution was cast onto an aluminum substrate using a 100 μm film casting knife, followed by the application of 0.5 mL of the top solution using a 150 μm casting knife. The film was then subjected to directional solidification by placing the aluminum plate on a −30 °C cold stage for 1 min, followed by immersion in ice water for 10 min. The resulting film was vacuum-dried at 2 Pa for 12 h to obtain the final PVDF/MXene composite film. Both the PVDF/MXene and nylon films were trimmed into 4 × 4 cm^2^ sheets, and copper tape was applied to serve as electrodes. These films were then affixed to acrylic substrates of matching size using double-sided adhesive tape. The electrical output performance of the assembled TENG was evaluated using a motor-driven crank-link mechanism.

### 4.6 Preparation of microneedle

PEGDA exhibits high stiffness and rapid photopolymerization properties and is widely used in microneedle fabrication [55]. Compared with natural polymers (e.g., alginate, gelatin, hyaluronic acid), employing PEGDA as the hydrogel microneedle base enables high-precision molding via fast UV-initiated crosslinking. Although PEGDA-based microneedles exhibit limited biodegradability, this limitation is suitable for microneedles with an electrical stimulation-responsive property [56]. First, a prepolymer solution consisting of 80 % (v/v) PEGDA with 2 % (v/v) Darocur 1173 was injected into a microneedle mold. Vacuum degassing was applied to ensure complete filling of both the needle body and base regions. The filled mold was then exposed to UV light to initiate crosslinking and form a uniform PEGDA microneedle patch. After demolding, the contact surface between the microneedles and the mold was marked to facilitate alignment during subsequent assembly. To impart electrical conductivity, a platinum layer (∼100 nm thick) was deposited onto the microneedle surface via magnetron sputtering (ULVAC, ACS-4000-C4). For the construction of the drug-loading layer, a homogeneous precursor solution was prepared by dissolving PEGDA (12 % v/v), GelMA (16 % v/v), and photoinitiator (0.25 % w/v), followed by the addition of KMD@AsEXO PBS solution (5 mg/ml). This solution was reinjected into the original mold, and vacuum pumping was used to fill only the needle tip region. Excess solution was carefully removed, and a custom-fabricated 200 μm-thick metal substrate was placed on the mold. The prefabricated single-layer conductive microneedles were then aligned and pressed into position. A second round of UV curing was performed to complete the formation of the double-layer microneedle structure. Finally, the assembled microneedle patch was dried in a 37 °C oven for 24 h to obtain a structurally complete, drug-loaded, double-layer conductive microneedle patch.

### 4.7 Assembly of the self-powered drug delivery microneedle platform

The flexible printed circuit board was designed using EDA software, featuring electrode pads on both the top and bottom surfaces, interconnected via plated through-holes (Fig. S4). This configuration enables electrical signals transmitted through external wiring to be efficiently routed to the bottom electrodes. To facilitate stable electrical contact and mechanical integration with the microneedle array, a custom aluminum alloy base was fabricated using metal 3D printing (Fig. S3). The base was precisely dimensioned to accommodate the microneedle patch and ensure reliable positioning. For final assembly, the single-layer conductive microneedle array was adhered to the negative electrode of the flexible printed circuit board using conductive adhesive. The double-layer, drug-loaded conductive microneedle array was placed on the positive electrode. Two wires extending from the FPCB’s top surface were then connected to the TENG output via a bridge rectifier circuit, enabling direct current stimulation for controlled drug release.

### 4.8 Characterization

Proton nuclear magnetic resonance (1H NMR, Bruker Plus, 600 MHz) was used to characterize the chemical structure of KRWWKWWRRC-PEG-DSPE. Fourier-transform infrared spectroscopy (FTIR, TA Instruments, TGA-Q500) was employed to analyze functional group changes among EXO, AsEXO, and KMD@AsEXO samples. A laser particle size and potential analysis measurement system (MS-2000, Malvern) was used to determine particle size distribution and surface charge. The morphological features of exosomes were observed using transmission electron microscopy (TEM, Tecnai G2, Thermo Fisher Scientific) after negative staining with phosphotungstic acid. Protein expression in exosome samples was assessed via standard western blotting procedures, including SDS-PAGE electrophoresis, membrane transfer, and antibody incubation. The electrical output of the TENG was measured using an electrostatic voltmeter (Keithley 6517A) equipped with a signal acquisition card. The mechanical properties of the microneedle patches were evaluated using a universal testing machine (WDW-5Y) at a loading speed of 2 mm/min.

### 4.9 Drug release

The microneedle module was placed into a custom-designed 3D-printed mold. The needle tips were immersed in 2 mL of PBS buffer within the mold, which was maintained in a 37 °C water bath to simulate physiological conditions. KMD@AsEXO were pre-labeled with PKH26 fluorescent dye. The TENG device was operated at a frequency of 4 Hz to provide pulsed electrical stimulation. At predetermined time points, 100 μL of the release medium was collected for analysis. Fluorescence intensity was measured using a fluorescence spectrophotometer (F97Pro, Lengguang) with an excitation wavelength of 551 nm and an emission wavelength of 567 nm. After each measurement, 100 μL of fresh PBS was added to maintain a constant volume.

### 4.10 KMD@AsEXO uptake anysis

All exosome samples used in the self-powered microneedle system were pre-labeled with PKH26. Using the previously described drug-release molds, 2 mL of cell culture medium was added under sterile conditions. Drug release was conducted for 24 h under two conditions: with electrical stimulation and without electrical stimulation. Subsequently, HUVECs were seeded into 24-well plates and incubated with the collected release media for an additional 24 h. After incubation, cells were fixed and stained for both the cytoskeleton and nuclei. Cellular uptake of the released exosomes was then visualized using a fluorescence microscope (Axio Zoom.V16, Zeiss).

### 4.11 *In vitro* antibacterial and anti-biofilm evaluation

*E. coli* and *S. aureus* were cultured in LB medium at 37 °C with constant shaking. All experiments were performed using bacteria in the logarithmic growth phase. The bacterial suspension was then adjusted to a concentration of 10^6^ CFU/mL using normal saline, and a 3D-printed mold designed for drug release was introduced. The control group received no treatment. In the TENG group, the needle tips of the assembled microneedle module without exosome drugs were applied to the bacterial solution and subjected to 4 Hz electrical stimulation for 24 h. In the KMD@AsEXO group, drug release occurred over 24 h without electrical stimulation. In the KMD@AsEXO/TENG group, the assembled microneedle module was electrically stimulated by the TENG at 4 Hz for 24 h. After treatment, the bacterial suspensions were serially diluted 100-fold, and 100 μL suspensions were spread onto LB agar plates, followed by incubation at 37 °C for 24 h in a bacterial culture chamber (Thermo Fisher). The number of viable colonies was counted. Additionally, to assess bacterial viability, the suspensions were concentrated 100-fold, stained using a bacterial live/dead staining kit, and visualized using a confocal laser scanning microscope (CLSM, FV3000, Olympus).

To evaluate the *in vitro* antibiofilm efficacy of the self-powered microneedle delivery platform loaded with KMD@AsEXO, a crystal violet staining assay was performed. Briefly, 2 mL of pancreatic protein-dextrose medium containing *S. aureus* at a concentration of 10^6^ CFU/mL was added to each well of a 24-well plate. The two types of microneedles were vertically inserted into the culture medium along the inner wall of each well in the 24-well plate. In the KMD@AsEXO/TENG group, the TENG was connected to provide electrical stimulation at a frequency of 4 Hz for 48 h. Following incubation, the supernatant was removed, and the wells were washed with PBS to eliminate planktonic bacteria. Each well was then incubated with 1 ml of 0.05 % crystal violet solution for 10 min. After discarding the dye, the wells were rinsed thoroughly with distilled water. The retained crystal violet-stained biofilm was solubilized using 1 mL of 30 % (v/v) acetic acid, and the absorbance at 570 nm was measured using a microplate reader to quantify biofilm biomass. The KMD@AsEXO group underwent the same procedure without electrical stimulation. The TENG group utilized double-layer microneedles without KMD@AsEXO, connected to the TENG for electrical output. The control group received no treatment. For CLSM imaging, the procedure was similar. A sterile glass coverslip was placed in each well before the addition of the bacterial culture medium. Each group followed the same treatment conditions as described above. After incubation and PBS washing, the biofilms in the glass coverslip were stained with SYTO 9 for 30 min and imaged using a CLSM (FV3000, Olympus).

### 4.12 Hemocompatibility

To assess hemocompatibility, the drug-loaded double-layer microneedles were extracted using normal saline at a ratio of 0.2 g/mL at 37 °C for 24 h to obtain the extract solution. RBCs were suspended in the extract (5 % v/v) and exposed to electrical stimulation from the TENG at 4 Hz for 1 h, forming the KMD@AsEXO/TENG group. For the control group, RBCs suspended in deionized water and normal saline served as the positive and negative control groups, respectively. In the TENG group, RBCs were suspended in normal saline and connected to the TENG for 1 h. The KMD@AsEXO group involved RBCs suspended in the extract (5 % v/v) without electrical stimulation for 1 h. All experiments were conducted at 37 °C. After treatment, samples were centrifuged at 800×*g*, and the absorbance of the supernatant was measured at 545 nm.

### 4.13 Cell proliferation experiment

NIH-3T3 cells were seeded into a 24-well plate at a density of 9000 cells per well in complete medium and incubated for 6 h to allow for cell adhesion. Following attachment, two types of microneedles (conductive single-layer microneedles and drug-loaded conductive double-layer microneedles) were inserted vertically into the medium. For the KMD@AsEXO/TENG group, the two types of microneedles were connected to the TENG to deliver electrical stimulation at a frequency of 4 Hz for 24 h. The control group received no treatment. The KMD@AsEXO group underwent the same procedure without electrical stimulation, while the TENG group received electrical stimulation without the addition of KMD@AsEXO.

### 4.14 Cell scratch assay

When NIH-3T3 cells reached full confluency in the culture dish, a linear scratch was created using a sterile pipette tip. The culture medium was then replaced with medium containing 2 % serum. For the KMD@AsEXO/TENG group, the conductive single-layer microneedles and drug-loaded conductive double-layer microneedles were immersed in the medium through the lid of the culture dish and connected to the TENG operating at 4 Hz for 24 h. The direction of the applied electric field was aligned with the direction of cell migration. The control group received no treatment. The KMD@AsEXO group was treated without electrical stimulation, while the TENG group was treated without KMD@AsEXO. After 24 h of treatment, cell migration was observed and imaged under a microscope, and the migration rate was quantified.

### 4.15 Anti-inflammatory assay

RAW264.7 cells were seeded into 24-well plates and stimulated with 100 ng/mL lipopolysaccharide (LPS) for 24 h to induce an inflammatory response. Conductive single-layer microneedles and drug-loaded conductive double-layer microneedles were immersed in the culture medium via the lid of the culture dish and connected to the TENG, delivering electrical stimulation at 4 Hz. After 24 h of treatment, this group was designated as the KMD@AsEXO/TENG group. The control group received no treatment. The KMD@AsEXO group was treated without electrical stimulation, while the TENG group received electrical stimulation without the KMD@AsEXO. Following treatment, the cell culture supernatant was collected by centrifugation at 2000×*g* for 10 min. The levels of inflammatory cytokines were then quantified using enzyme-linked immunosorbent assay (ELISA) kits, according to the manufacturer’s instructions.

### 4.16 *In vivo* evaluation of infected wound healing

All animal experiments were approved by the Ethics Committee of the Life Science Center at Harbin Institute of Technology (Approval No. IACUC-2023056) and conducted according to institutional animal welfare guidelines. Ten-week-old male Balb/c mice were randomly divided into five groups (n = 3 per group): Control, TENG, TENG/EXO, KMD@AsEXO, and KMD@AsEXO/TENG. Mice were housed under standard conditions (12-h light/dark cycle) and provided with ad libitum access to standard chow. After depilation using a hair removal device and cream, a full-thickness skin wound model was established by a 6 mm hole punch on the back skin. Wounds in each group were infected with *S.aureus* suspension (20 μL, 10^8^ CFU/mL). The assembled microneedle module was applied to the wound site and connected to the TENG (24 h, 4 Hz). Then, they were covered with medical film (Tegaderm, 3M, 1626 W) as the KMD@AsEXO/TENG group. Microneedle patches were replaced every three days. The control group was only covered with medical film; the TENG group received electrical stimulation with drug-free double-layer microneedles; the TENG/EXO group received electrical stimulation with free exosome-loaded double-layer microneedles; the KMD@AsEXO group was treated with drug-loaded microneedles without electrical stimulation. On day 12, mice were euthanized, and wound tissues were harvested and fixed in 4 % paraformaldehyde overnight. Histological analyses were performed using hematoxylin and eosin (H&E) staining and Masson’s trichrome staining. Quantitative analysis of the stained sections was conducted using ImageJ software. Besides, fifteen wound models were also established and assigned to five groups (n = 3 per group), treated as described above. On day 6, skin tissue encompassing the entire wound area was collected from each mouse and homogenized in 1 mL PBS. The homogenate was diluted 1:100 in PBS, and 100 μL of the diluted sample was spread onto LB agar plates. After 24 h incubation, plates were imaged, and colony-forming units were counted. For the *in vivo* biocompatibility assessment, six mice were randomly assigned to two groups (n = 3 per group): a healthy control group (no treatment) and an infected-wound model group (wound creation followed by bacterial inoculation). The infected group received KMD@AsEXO/TENG treatment as mentioned before on day 0 and then every three days for a total of seven sessions (days 0, 3, 6, 9, 12, 15, and 18). On day 20, mice were anesthetized with isoflurane, blood was collected via the retro-orbital blood collection, and analyzed using a Veterinary Automatic Biochemistry Analyzer (MSLDA22). The heart, liver, spleen, lungs, and kidneys were then harvested for hematoxylin–eosin (H&E) staining.

### 4.17 Data analysis

All experiments were independently repeated at least three times. Data are presented as mean ± standard deviation (mean ± SD). One-way analysis of variance (ANOVA) was performed using SPSS 27 to compare means among multiple groups. A p-value of less than 0.05 was considered statistically significant. Significance levels are indicated in the figures as follows: ns (not significant), ∗ (p < 0.05), ∗∗ (p < 0.01), ∗∗∗ (p < 0.001), and ∗∗∗∗ (p < 0.0001).

## CRediT authorship contribution statement

**Shanguo Zhang:** Writing – original draft, Validation, Formal analysis, Data curation. **Tianyi Jiang:** Writing – review & editing, Writing – original draft, Software, Resources, Project administration, Methodology, Investigation. **Depeng Yang:** Investigation. **Liangyu Cao:** Investigation. **Ming Li:** Investigation. **Jiachao Tang:** Investigation. **Qi Gu:** Investigation. **Aitong Xu:** Investigation. **Yu Li:** Writing – original draft, Validation, Investigation. **Hongyuan Jiang:** Writing – review & editing, Writing – original draft, Methodology, Funding acquisition.

## Declaration of competing interest

The authors declare that they have no competing interests.

## Data Availability

Data will be made available on request.

## References

[1] Isei T., Abe M., Ikegami R., Kato H., Sakurai E., Tanizaki H., Nakanishi T., Matsuo K., Yamasaki O., Asai J. (2025). Wound, pressure ulcer, and burn guidelines–3: guidelines for the diagnosis and treatment of diabetic ulcers and gangrene. J. Dermatol..

[2] Vo V., Haidari H., Cowin A.J., Wagstaff M., Dearman B., Kopecki Z. (2025). Dermal substitutes for clinical management of severe burn injuries: current and future perspectives. Adv. Ther..

[3] Chen X., Zhang Y., Yu W., Zhang W., Tang H., Yuan W.-E. (2023). In situ forming ROS-scavenging hybrid hydrogel loaded with polydopamine-modified fullerene nanocomposites for promoting skin wound healing. J. Nanobiotechnol..

[4] Zhang Y., Kang J., Chen X., Zhang W., Zhang X., Yu W., Yuan W.-E. (2023). Ag nanocomposite hydrogels with immune and regenerative microenvironment regulation promote scarless healing of infected wounds. J. Nanobiotechnol..

[5] Hurlow J., Wolcott R.D., Bowler P.G. (2025). Clinical management of chronic wound infections: the battle against biofilm. Wound Repair Regen..

[6] Uberoi A., McCready-Vangi A., Grice E.A. (2024). The wound microbiota: microbial mechanisms of impaired wound healing and infection. Nat. Rev. Microbiol..

[7] Xiang G., Wang B., Zhang W., Dong Y., Tao J., Zhang A., Chen R., Jiang T., Zhao X. (2024). A Zn-MOF-GOx-based cascade nanoreactor promotes diabetic infected wound healing by NO release and microenvironment regulation. Acta Biomater..

[8] Zhang X., Yu W., Zhang Y., Zhang W., Wang J., Gu M., Cheng S., Ren G., Zhao B., Yuan W.-E. (2024). A hydrogen generator composed of poly (lactic-co-glycolic acid) nanofibre membrane loaded iron nanoparticles for infectious diabetic wound repair. J. Colloid Interface Sci..

[9] Wang Z., Liang X., Wang G., Wang X., Chen Y. (2025). Emerging bioprinting for wound healing. Adv. Mater..

[10] Sun Y., Liu M., Sun W., Tang X., Zhou Y., Zhang J., Yang B. (2024). A hemoglobin bionics‐based System for combating antibiotic resistance in chronic diabetic wounds via iron homeostasis regulation. Adv. Mater..

[11] Toufanian S., Mohammed J., Winterhelt E., Lofts A., Dave R., Coombes B.K., Hoare T. (2024). A nanocomposite dynamic covalent cross-linked hydrogel loaded with fusidic acid for treating antibiotic-resistant infected wounds. ACS Appl. Bio Mater..

[12] Tang H., Qu X., Zhang W., Chen X., Zhang S., Xu Y., Yang H., Wang Y., Yang J., Yuan W.-e., Yue B. (2022). Photosensitizer nanodot eliciting immunogenicity for photo-immunologic therapy of postoperative Methicillin-resistant Staphylococcus aureus infection and secondary recurrence. Adv. Mater..

[13] Zhang W., Wang Y., Zhang X., Zhang Y., Yu W., Tang H., Yuan W.-E. (2025). Polyzwitterion-branched polycholic acid nanocarriers based oral delivery insulin for long-term glucose and metabolic regulation in diabetes mellitus. J. Nanobiotechnol..

[14] Nuccitelli R. (2003). A role for endogenous electric fields in wound healing. Curr. Top. Dev. Biol..

[15] Luo R., Dai J., Zhang J., Li Z. (2021). Accelerated skin wound healing by electrical stimulation. Adv. Healthcare Mater..

[16] Zhang S., Jiang T., Han F., Cao L., Li M., Ge Z., Sun H., Wu H., Wu W., Zhou N., Akhtar M.L., Jiang H. (2024). A wearable self-powered microneedle system based on conductive drugs for infected wound healing: a new electrical stimulation delivery strategy. Chem. Eng. J..

[17] Cheng T., Shao J., Wang Z.L. (2023). Triboelectric nanogenerators. Nat. Rev. Methods Prim..

[18] Vivekananthan V., Kim W.J., Alluri N.R., Purusothaman Y., Abisegapriyan K., Kim S.J. (2019). A sliding mode contact electrification based triboelectric-electromagnetic hybrid generator for small-scale biomechanical energy harvesting. Micro Nano Syst. Lett..

[19] Zhou X., Li G., Wu D., Liang H., Zhang W., Zeng L., Zhu Q., Lai P., Wen Z., Yang C., Pan Y. (2023). Recent advances of cellular stimulation with triboelectric nanogenerators. Exploration.

[20] Han L., Li K., Wang Z., Men W., Wu X., Sun X., Zhang J., Cheng J. (2025). 3D printing flexible wearable electronics with diversified environmentally adaptive for biomechanical energy harvesting and personal electromagnetic safety. Adv. Funct. Mater..

[21] Luo W., Luo R., Liu J., Li Z., Wang Y. (2024). Self‐powered electrically controlled drug release systems based on nanogenerator. Adv. Funct. Mater..

[22] Zhang W., Qin X., Li G., Zhou X., Li H., Wu D., Song Y., Zhao K., Wang K., Feng X., Tan L., Wang B., Sun X., Wen Z., Yang C. (2024). Self-powered triboelectric-responsive microneedles with controllable release of optogenetically engineered extracellular vesicles for intervertebral disc degeneration repair. Nat. Commun..

[23] Gan N., Li X., Wei M., Li Z., Zhou S., Gao B. (2025). Tongue prick bionic angularly adjustable microneedles for enhanced scarless wound healing. Adv. Funct. Mater..

[24] Liu Y., Luo X., Li L., Chen L., Qiao Z., Si C., Haiyan J., Liu X. (2025). Wearable, battery-free, and wireless microneedle-based bioelectronics for robustly-integrated chronic wound management and therapeutic diagnosis. Nano Energy.

[25] Wang K., Ding Q., Qi M., Zhang W., Hou Y., Cao R., Li C., Xu L., Wang L., Kim J.S. (2024). Integrated bilayer microneedle dressing and triboelectric nanogenerator for intelligent management of infected wounds. Adv. Funct. Mater..

[26] Zhang S., Jiang T., Han F., Cao L., Li M., Ge Z., Sun H., Wu H., Wu W., Zhou N. (2024). A wearable self-powered microneedle system based on conductive drugs for infected wound healing: a new electrical stimulation delivery strategy. Chem. Eng. J..

[27] Zhang W., Qin X., Li G., Zhou X., Li H., Wu D., Song Y., Zhao K., Wang K., Feng X. (2024). Self-powered triboelectric-responsive microneedles with controllable release of optogenetically engineered extracellular vesicles for intervertebral disc degeneration repair. Nat. Commun..

[28] You W., Cai Z., Xiao F., Zhao J., Wang G., Wang W., Chen Z., Hu W., Chen Y., Wang Z. (2025). Biomolecular microneedle initiates Fe3O4/MXene heterojunction-mediated nanozyme-like reactions and bacterial ferroptosis to repair diabetic wounds. Adv. Sci..

[29] Saleem M., Shahzad K.A., Marryum M., Singh S., Zhou Q., Du S., Wang S., Shao C., Shaikh I.I. (2024). Exosome-based therapies for inflammatory disorders: a review of recent advances. Stem Cell Res. Ther..

[30] Zha X. (2025). Exosome-based therapy for spinal cord injury: a narrative review. Adv. Technol. Neurosci..

[31] Hu W., Wang W., Chen Z., Chen Y., Wang Z. (2024). Engineered exosomes and composite biomaterials for tissue regeneration. Theranostics.

[32] Yang J., Li Y., Jiang S., Tian Y., Zhang M., Guo S., Wu P., Li J., Xu L., Li W., Wang Y., Gao H., Huang Y., Weng Y., Ruan S. (2025). Engineered brain-targeting exosome for reprogramming immunosuppressive microenvironment of glioblastoma. Exploration.

[33] Liao H.J., Yang Y.P., Liu Y.H., Tseng H.C., Huo T.I., Chiou S.H., Chang C.H. (2024). Harnessing the potential of mesenchymal stem cells–derived exosomes in degenerative diseases. Regener. Ther..

[34] Jiao Y.R., Chen K.X., Tang X., Tang Y.L., Yang H.L., Yin Y.L., Li C.J. (2024). Exosomes derived from mesenchymal stem cells in diabetes and diabetic complications. Cell Death Dis..

[35] Odunitan T.T., Apanisile B.T., Afolabi J.A., Adeniwura P.O., Akinboade M.W., Ibrahim N.O., Alare K.P., Saibu O.A., Adeosun O.A., Opeyemi H.S. (2025). Beyond conventional drug design: exploring the broad‐spectrum efficacy of antimicrobial peptides. Chem. Biodiversity.

[36] Yadav N., Chauhan V.S. (2024). Advancements in peptide-based antimicrobials: a possible option for emerging drug-resistant infections. Adv. Colloid Interface Sci..

[37] Qu X., Wang M., Wang M., Tang H., Zhang S., Yang H., Yuan W., Wang Y., Yang J., Yue B. (2022). Multi-mode antibacterial strategies enabled by gene-transfection and immunomodulatory nanoparticles in 3D-Printed scaffolds for synergistic exogenous and endogenous treatment of infections. Adv. Mater..

[38] Razif R., Fadilah N.I.M., Ahmad H., Looi Qi Hao D., Maarof M., Fauzi M.B. (2025). Asiaticoside-loaded multifunctional bioscaffolds for enhanced hyperglycemic wound healing. Biomedicines.

[39] Mu X., Chen J., Zhu H., Deng J., Wu X., He W., Ye P., Gu R., Wu Y., Han F. (2025). Asiaticoside-nitric oxide synergistically accelerate diabetic wound healing by regulating key metabolites and SRC/STAT3 signaling. Burn. Trauma.

[40] Kumar M., Kumar D., Mahmood S., Singh V., Chopra S., Hilles A.R., Bhatia A. (2024). Nanotechnology-driven wound healing potential of asiaticoside: a comprehensive review. RSC Pharm..

[41] Huang H., Xie Y., Zhong J., Fu Z., Wu P., Chen X., Xiao Z., Yuan J., Shi X., Liang D. (2024). Antimicrobial peptides loaded collagen nanosheets with enhanced antibacterial activity, corneal wound healing and M1 macrophage polarization in bacterial keratitis. Composites, Part B.

[42] Yuan Z., Kang J., Wu S., Dong A., Wu R. (2025). Advances in antimicrobial peptide‐based biomaterials for combating multidrug‐resistant bacterial infections. Macromol. Rapid Commun..

[43] Jing B., Gai Y., Qian R., Liu Z., Zhu Z., Gao Y., Lan X., An R. (2021). Hydrophobic insertion-based engineering of tumor cell-derived exosomes for SPECT/NIRF imaging of colon cancer. J. Nanobiotechnol..

[44] Kooijmans S.A.A., Fliervoet L.A.L., van der Meel R., Fens M.H.A.M., Heijnen H.F.G., van Bergen en Henegouwen P.M.P., Vader P., Schiffelers R.M. (2016). PEGylated and targeted extracellular vesicles display enhanced cell specificity and circulation time. J. Contr. Release.

[45] Zhang S., Jiang T., Li M., Sun H., Wu H., Wu W., Li Y., Jiang H. (2024). Multifunctional wearable triboelectric nanogenerators prepared by combined crystallization and diffusion method: high-output, breathable, antimicrobial, and Janus-wettability for smart control and self-powered biosensing. Nano Energy.

[46] Kong L., Tang M., Zhang Z., Pan Y., Cao H., Wang X., Ahmed A. (2022). A near-zero energy system based on a kinetic energy harvester for smart ranch. iScience.

[47] Li G., Wu H., Guo R., Zhang H., Li L., Iqbal M.Y., Gu F. (2022). A triboelectric piston–cylinder assembly with condition‐monitoring and self‐powering capabilities. Energy Technol..

[48] Lei Q., He D., Ding L., Kong F., He P., Huang J., Guo J., Brinker C.J., Luo G., Zhu W. (2022). Microneedle patches integrated with biomineralized melanin nanoparticles for simultaneous skin tumor photothermal therapy and wound healing. Adv. Funct. Mater..

[49] Waghule T., Singhvi G., Dubey S.K., Pandey M.M., Gupta G., Singh M., Dua K. (2019). Microneedles: a smart approach and increasing potential for transdermal drug delivery system. Biomed. Pharmacother..

[50] Öztürk K., Kaplan M., Çalış S. (2024). Effects of nanoparticle size, shape, and zeta potential on drug delivery. Int. J. Pharm..

[51] Waknis V., S, Jonnalagadda (2011). Novel poly-DL-lactide-polycaprolactone copolymer based flexible drug delivery system for sustained release of ciprofloxacin. Drug Deliv..

[52] Gagandeep K., Narasingappa R.B., Vyas G.V. (2024). Unveiling mechanisms of antimicrobial peptide: actions beyond the membranes disruption. Heliyon.

[53] Gagat P., Ostrówka M., Duda-Madej A., Mackiewicz P. (2024). Enhancing antimicrobial peptide activity through modifications of charge, hydrophobicity, and structure. Int. J. Mol. Sci..

[54] Li J., Koh J.-J., Liu S., Lakshminarayanan R., Verma C.S., Beuerman R.W. (2017). Membrane active antimicrobial peptides: translating mechanistic insights to design. Front. Neurosci..

[55] Gao Y., Hou M., Yang R., Zhang L., Xu Z., Kang Y., Xue P. (2018). PEGDA/PVP microneedles with tailorable matrix constitutions for controllable transdermal drug delivery. Macromol. Mater. Eng..

[56] Chen Z., Hu X., Lin Z., Mao H., Qiu Z., Xiang K., Ke T., Li L., Lu L., Xiao L. (2023). Layered GelMA/PEGDA hydrogel microneedle patch as an intradermal delivery system for hypertrophic scar treatment. ACS Appl. Mater. Interfaces.

