# Supplementary material for: Dual-domain engineered exosome-based self-powered microneedle delivery platform for the treatment of infected wounds
Source: Mater Today Bio. 2025 Oct 7;35:102383. doi: 10.1016/j.mtbio.2025.102383 (PMC12550190; doi:10.1016/j.mtbio.2025.102383)
Supplement: Multimedia component 1 [file mmc1.docx]

***Supporting information for***

**Dual-Domain Engineered Exosome-Based Self-Powered Microneedle Delivery Platform for the Treatment of Infected Wounds**

Shanguo Zhang ^a^, Tianyi Jiang ^a,*^, Depeng Yang ^b^, Liangyu Cao ^b^, Ming Li ^a^, Jiachao Tang ^a^, Aitong Xu, ^b^ Qi Gu ^b^, Yu Li ^b^, Hongyuan Jiang ^a^

^a^ School of Mechatronics Engineering, Harbin Institute of Technology, No.92 West Da-zhi Street, Harbin 150001, People’s Republic of China.

^b^ School of Life Sciences, Harbin Institute of Technology, No.2 Yikuang Street, Harbin 150001, People's Republic of China.

^*^ Corresponding author.

E-mail addresses:

jty_hit@hit.edu.cn (Tianyi Jiang)

**Table S1**. Comparative Analysis of Exosome-Based Therapies for Infected Wound Healing

| **Reference** | **Exosome Source** | **Delivery Strategy** | **Engineered** | **Antibacterial Mechanism** | **Active release** |
| --- | --- | --- | --- | --- | --- |
| Theranostics 2019; 9(1):65-76 | MSC | Injectable hydrogel | Yes | ε-Polylysine antimicrobial peptide | No |
| Journal of Materials Chemistry B 2020,8, 7197-7212 | MSC | Chitosan-silk fibroin dressing | Partial | Adsorbed AgNPs | No |
| Bioactive Materials, 2024, 42: 32-51. | Platelet | Dissolvable microneedle | No | None | No |
| Materials Today Bio, 2023, 20: 100649. | M2 Macrophage | Microneedle + photothermal | Partial | NIR-induced mild hyperthermia | Yes |
| International journal of nanomedicine, 2023: 949-970. | Keratinocyte | Injection | Yes | None | No |
| Journal of Nanobiotechnology 2023,21, 308 | Neutrophil-mimetic | Extracellular matrix-based hydrogel | Yes | Natural peroxidase/lysozyme | No |
| Journal of the American Chemical Society, 2025, 147, 19, 16362–16378 | MSC | Microenvironment-responsive hydrogel | Yes | Antibiotics | Yes |
| Signal Transduction and Targeted Therapy,2023, 8, 62 (2023). | Placental MSC | Silk fibroin patch | Yes | None | No |
| Journal of Controlled Release, 2025, 382: 113730. | MSC | Bacteria-responsive system | Yes | Antibiotics | Yes |
| Frontiers in Microbiology, 2025, 16: 1550276. | MSC | Exosome microspheres + AgNPs in hydrogel | Partial | AgNPs | No |
| Nature Communication,2024, 15, 3435. | MSC | Oxygen nanobubble-loaded hydrogel | No | None | No |
| Journal of nanobiotechnology, 2022, 20(1): 147. | HUVEC | Microneedle patch | Yes | None | No |
| Materials Today Bio, 2022, 16: 100365. | MSC | PEG hydrogel | Yes | None | No |
| Biomaterials, 2024, 308: 122558. | MSC | Hyaluronic acid hydrogel | No | Antimicrobial peptides | No |

**Table S2**. Blood routine examination of mice after 20 days of KMD@AsEXO/TENG treatment

| Parameters | Full title | Results | | Unit | Reference ranges |
| --- | --- | --- | --- | --- | --- |
|  |  | Control | KMD@AsE  XO/TENG |  |  |
| WBC | White blood cell count | 7.19 | 8.00 | 10^9^/L | 1.05 - 10.60 |
| Gran# | Absolute neutrophil count | 2.10 | 2.19 | 10^9^/L | 0.62 - 7.60 |
| Lym# | Absolute lymphocyte count | 4.59 | 5.37 | 10^9^/L | 0.90 - 10.60 |
| Mon# | Absolute monocyte count | 0.50 | 0.44 | 10^9^/L | 0.04 - 1.40 |
| Gran% | Neutrophil percentage | 29.2 | 27.4 | % | 10.5 - 59.0 |
| Lym% | Lymphocyte percentage | 63.8 | 67.1 | % | 35.0 - 83.0 |
| Mon% | Monocyte percentage | 7.0 | 5.5 | % | 0.0 - 10.2 |
| RBC | Red blood cell count | 10.54 | 10.33 | 10^12^/L | 6.50 - 11.50 |
| HGB | Hemoglobin | 162 | 162 | g/L | 110 - 165 |
| HCT | Hematocrit | 49.6 | 48.8 | % | 33.1 - 53.0 |
| MCV | Mean corpuscular volume | 47.1 | 47.2 | fL | 41.0 - 55.0 |
| MCH | Mean corpuscular hemoglobin | 15.3 | 15.7 | pg | 13.0 - 18.0 |
| MCHC | Mean corpuscular hemoglobin concentration | 326 | 332 | g/L | 300 - 360 |
| PLT | Platelet count | 604 | 792 | 10^9^/L | 400 - 2300 |
| MPV | Mean platelet volume | 6.4 | 6.7 | fL | 4.5 - 7.7 |
| PDW | Platelet distribution width | 15.7 | 15.7 |  | 12.0 - 17.5 |
| PCT | Plateletcrit | 0.389 | 0.528 | % | 0.250 - 1.250 |
| P-LCC | Platelet large cell count | 238 | 340 | 10^9^/L | 150 - 1000 |
| P-LCR | Platelet large cell ratio | 39.5 | 42.9 | % | 22.0 - 63.5 |


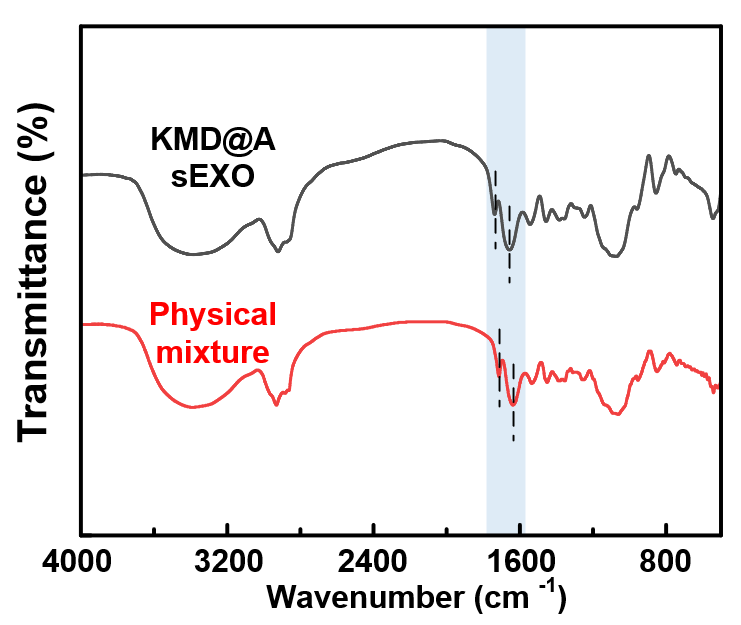


**Figure S1**. FTIR spectrum of KMD@AsEXO and solid-state physical mixture of KRWWKWWRRC–PEG–DSPE, asiaticoside, and exosomes.


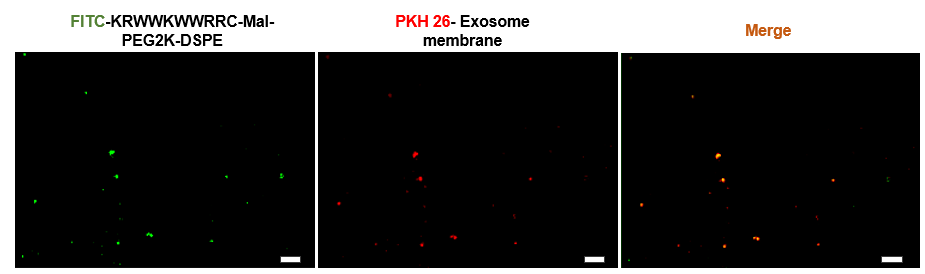


**Figure S2**. Confocal fluorescence images of KMD@AsEXO, with exosomal membranes labeled with PKH26 (red) and KRWWKWWRRC–PEG–DSPE labeled with FITC (green). Scale bars are 500 nm.


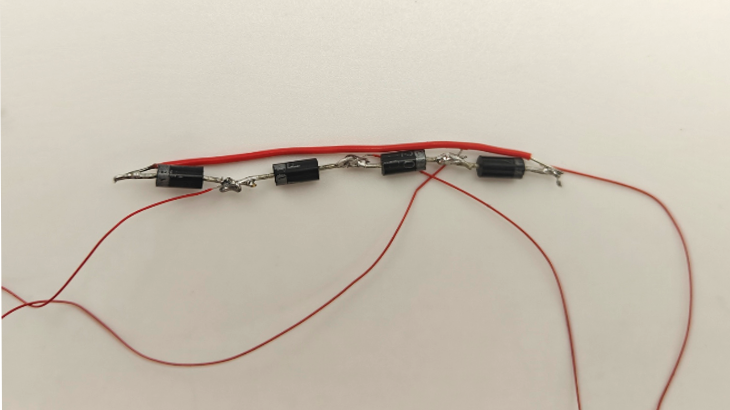


**Figure S3**. The rectifier bridge in the self-powered microneedle delivery platform.


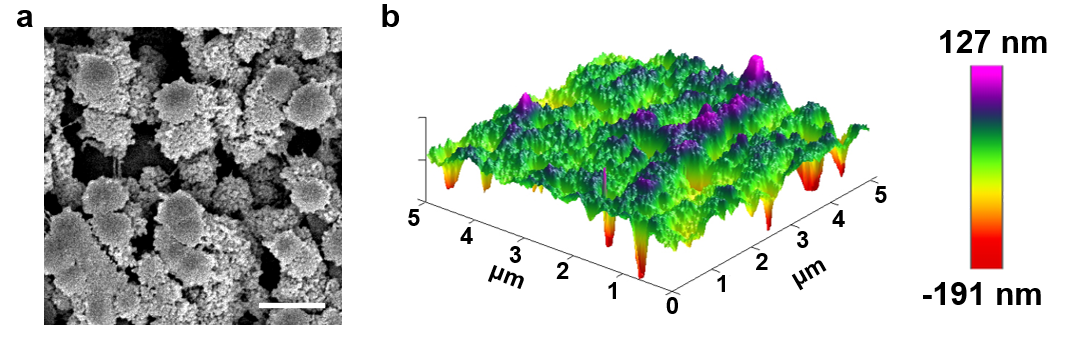


**Figure S4**. SEM (a) and atomic force microscope image (b) of PVDF/MXene composite membrane. The scale bar is 2 μm.


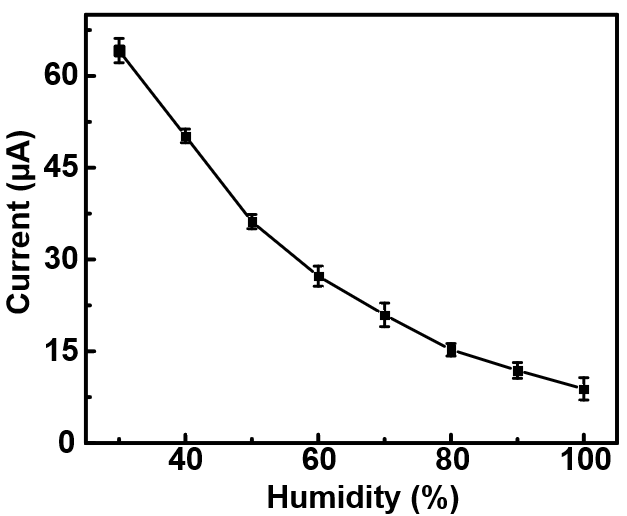


**Figure S5**. Humidity dependence of TENG current output


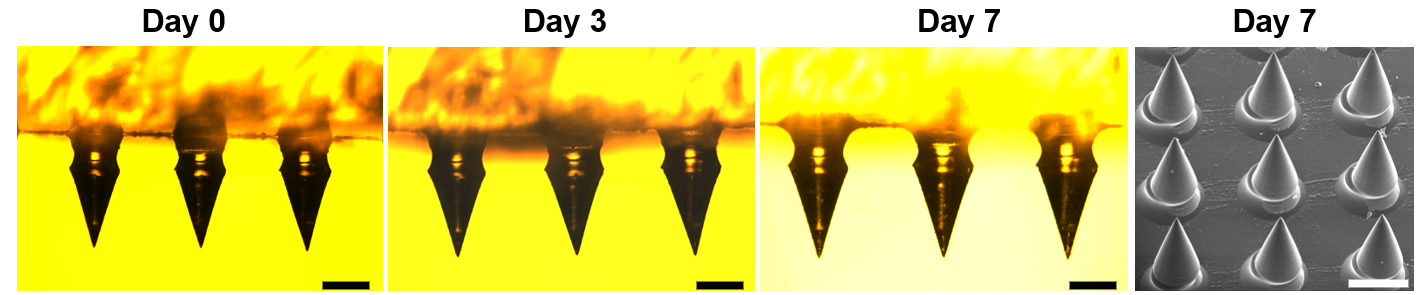


**Figure S6**. The stability of the microneedle tip in 37℃ PBS solution. All scale bars are 500 μm.


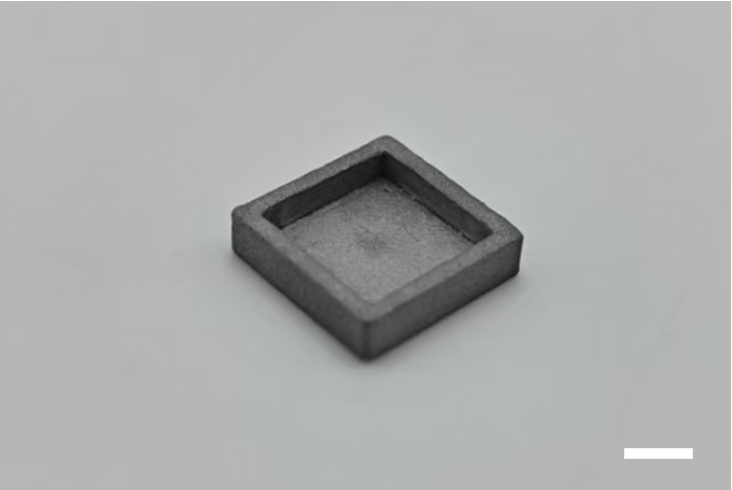


**Figure S7**. Image of the metal 3D-printed base used to connect the microneedle patch with the FPCB in the platform. The scale bar is 2 mm.


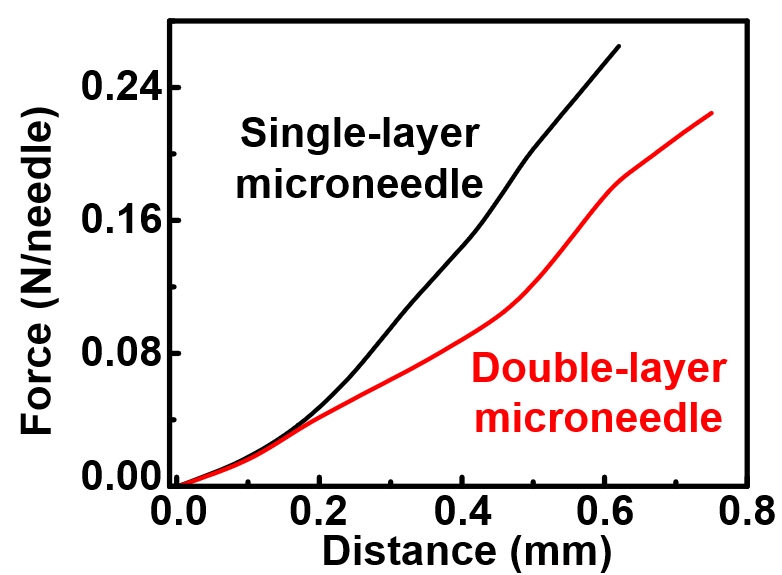


**Figure S8**. Force–displacement response of the microneedle under axial compression.


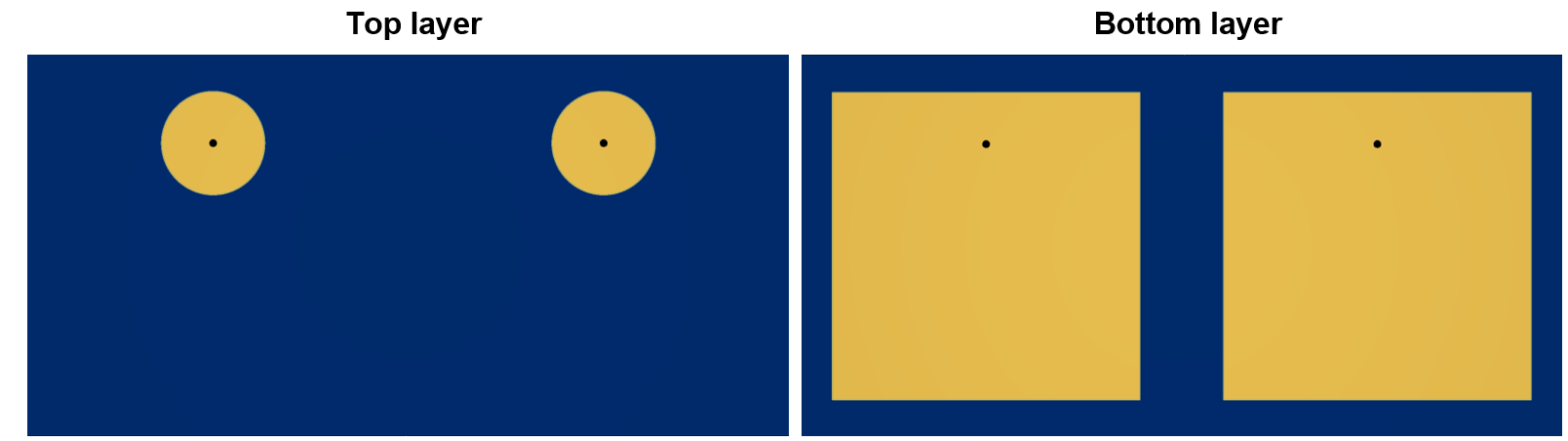


**Figure S9**. The electrodes on the top and bottom layers of the flexible circuit board are interconnected via through-holes.


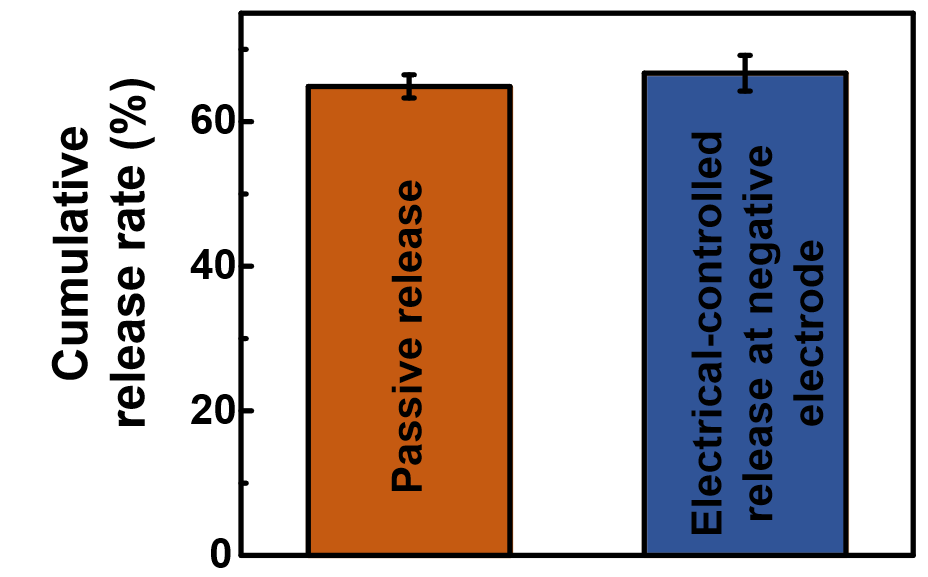


**Figure S10**. The drug release rate of the drug-loaded double-layer microneedle patch placed on the negative electrode of the flexible circuit board over 160 h.


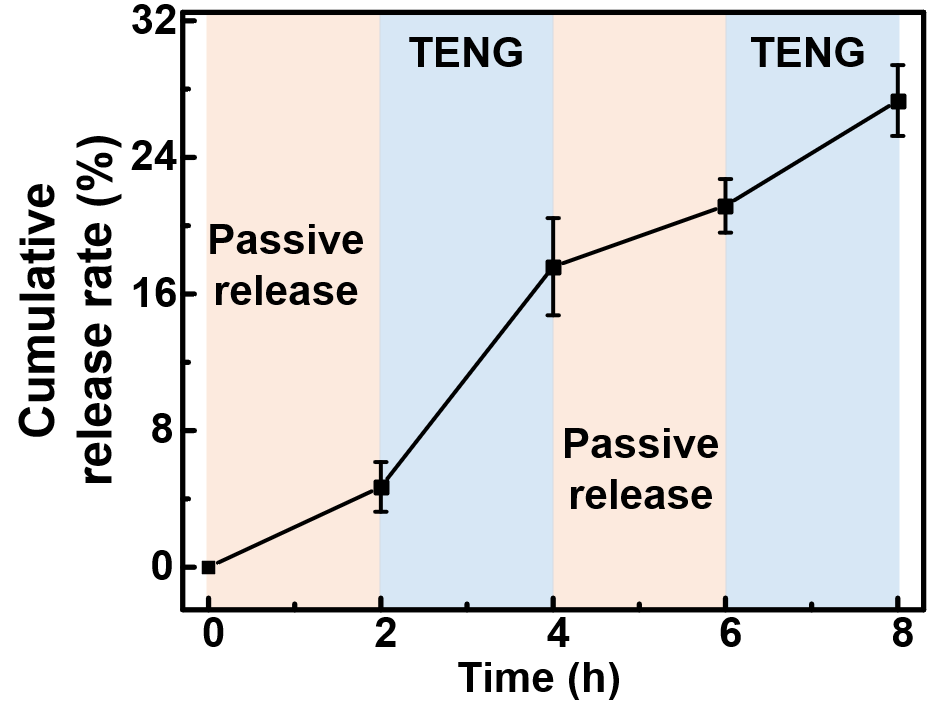


**Figure S11**. Intermittent drug release regulation using TENG.


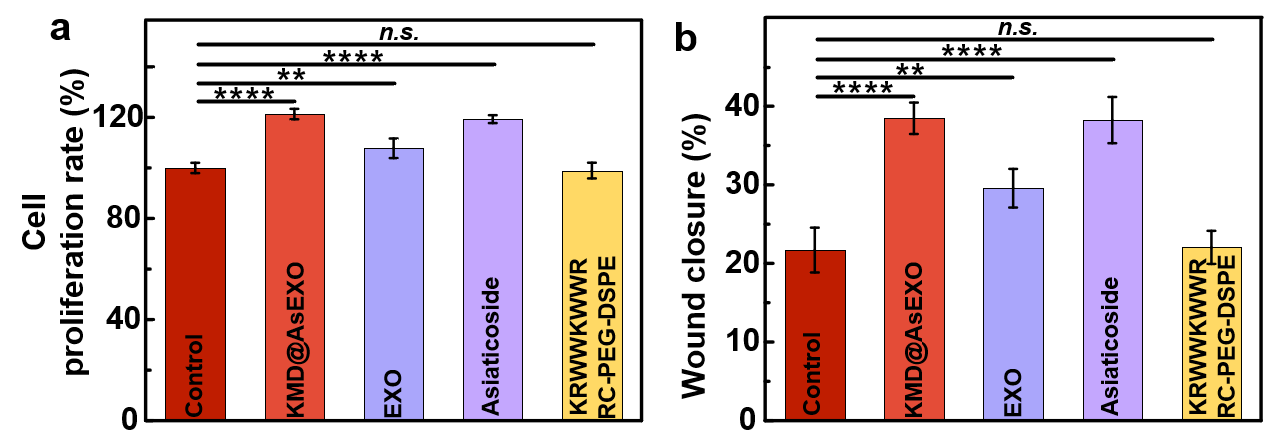


**Figure S12**. Effects of individual components in KMD@AsEXO on cell proliferation and migration.


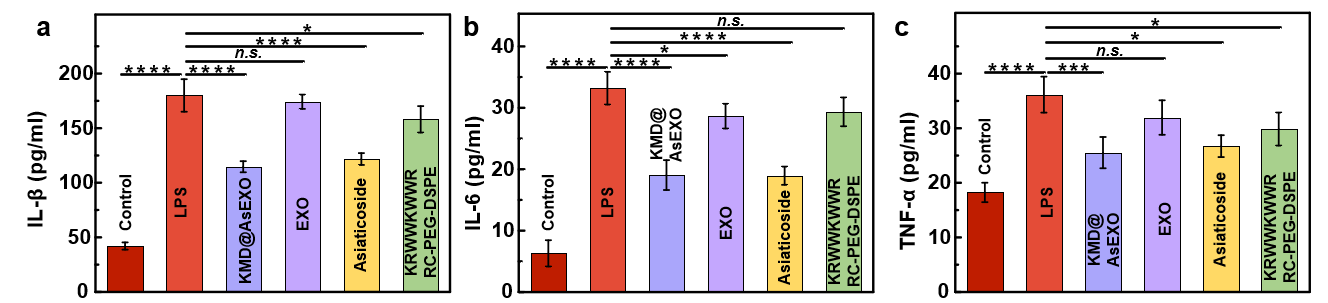


**Figure S13**. Effects of individual components in KMD@AsEXO on macrophage inflammatory responses.


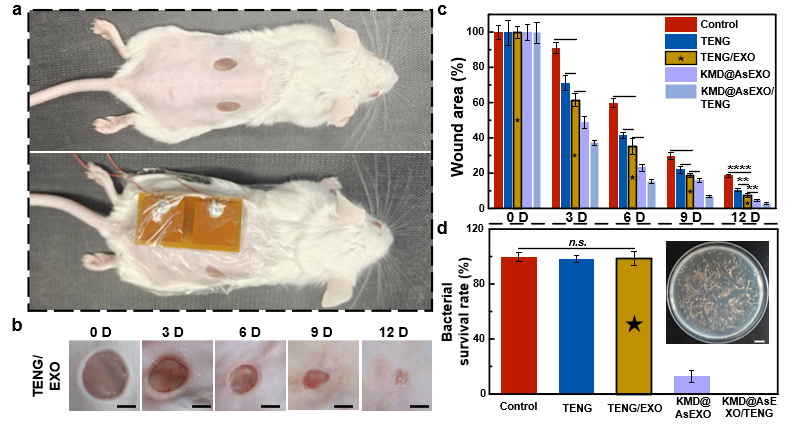


**Figure S14**. (a) Application of the KMD@AsEXO-loaded self-powered microneedle drug delivery platform for treating infected wounds in a mouse model. (b) Optical images of wound healing in the TENG/EXO group at designated time points (scale bar: 2 mm). (c) Quantitative analysis of wound area over time in the TENG/EXO group. (d) Bacterial survival was quantified by CFU counts from homogenized wound tissue in the TENG/EXO group on day 6. The inset shows representative bacterial agar plates for the corresponding groups.


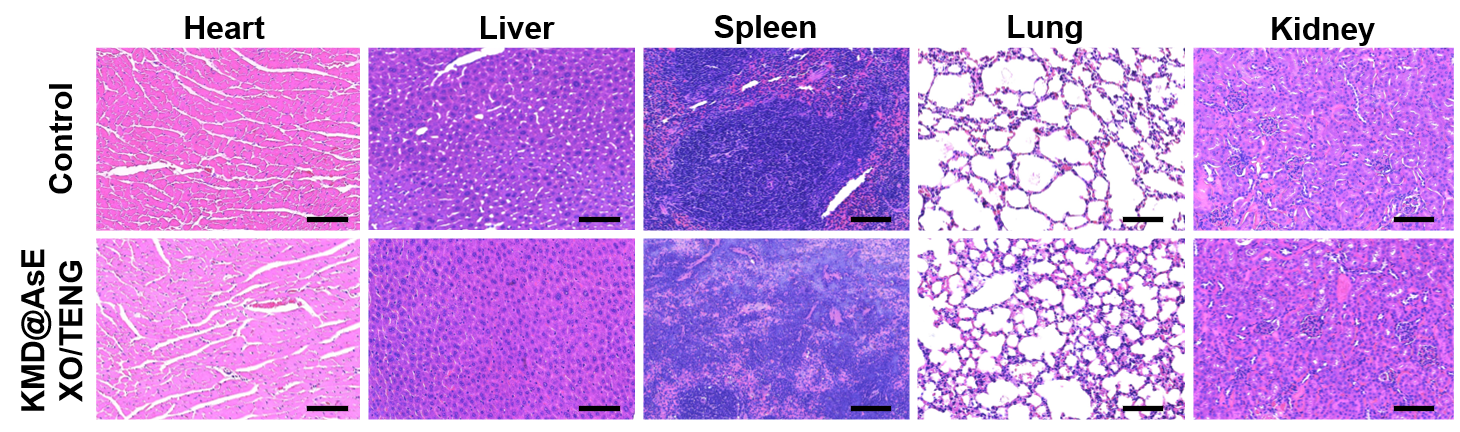


**Figure S15**. H&E staining of the heart, liver, spleen, lung, and kidney after treatment with KMD@AsEXO/TENG for 20 days. Scale bars are 100 μm.

**Note S1**:

We synthesized and characterized FITC–KRWWKWWRRC–PEG–DSPE. DSPE-PEG2K-Mal (5 mg) was dissolved in DMF (0.50 mL), then FITC-KRWWKWWRRCGK (1.1 equiv; obtained by SPPS from Shanghai Apeptide Co., Ltd.) and triethylamine (3.0 equiv) were added. The mixture was stirred at room temperature for 12 h (protected from light), transferred to a dialysis bag (MWCO 2,500 Da), dialyzed against deionized water for 24 h, and the retentate was lyophilized to afford the product. As shown in Figure N1, the FITC-conjugated sample displays additional multiplets in the aromatic region (δ 6.2–7.4 ppm) that are absent in the unlabeled group and coincide with the characteristic resonances of fluorescein, providing strong 1H-NMR evidence for successful formation of FITC–KRWWKWWRRC–PEG–DSPE.


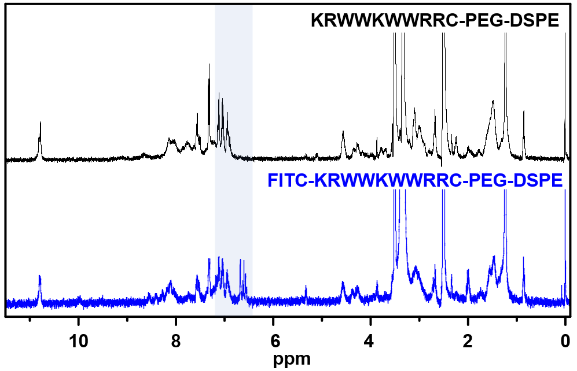


**Figure N1**. 1H NMR spectrum of FITC–KRWWKWWRRC–PEG–DSPE and KRWWKWWRRC–PEG–DSPE

**Note S2**:

The conductivity and resistance of the microneedle array were measured as follows. For conductivity, the assembled microneedle module was placed in a custom 3D-printed mold and immersed in 2 mL phosphate-buffered saline (PBS). The two top electrodes were connected to a benchtop conductivity meter (DDSJ-308F, LeiCi), and the stabilized reading was recorded. For DC resistance, the assembled module was laminated onto a 2.0 wt% agarose sheet pre-equilibrated with Ringer’s solution (147.2 mM NaCl, 4.02 mM KCl, 2.24 mM CaCl₂) with a thin silicone spacer. The top electrodes were wired to a resistance tester (SB2230, ShuangTe); All measurements were performed at room temperature.

In fabricating microneedles, GelMA was used as the structural matrix and PEGDA as the property-tuning material. To ensure the normal formation of the microneedles, GelMA was fixed at 16% (v/v): below ~15% reliable molding was difficult, whereas above ~20% the viscosity impeded mold filling and promoted bubble formation. It is also necessary to take into account the addition of PEGDA, so that the GelMA content does not become excessive. On this basis, PEGDA was varied across five levels (4, 8, 12, 16, 22%) to co-optimize mechanical strength and electrical conductivity, avoiding extreme, low-utility ratios. As PEGDA increased, crosslinking density rose and mechanical strength improved, while conductive pathways became restricted, leading to a gradual decrease in conductivity (Figure N2). Thus, considering both metrics identifies an optimal compromise at ~12% PEGDA.


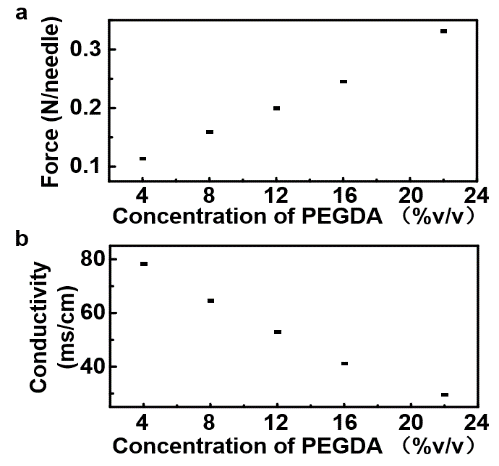


**Figure N2**. Mechanical strength (a) and electrical conductivity (b) of GelMA microneedles (GelMA 16% v/v) as a function of PEGDA content (4, 8, 12, 16, 22% v/v).

To balance conductivity and strength, we chose GelMA 16% (v/v) and PEGDA 12% (v/v) for the drug-loaded microneedles. With needle size fixed, electrical conductivity (σ) is inversely related to internal resistance( higher σ means lower R). The optimized resistance of the microneedle was measured to be approximately 52.7 kΩ.

**Note S3:**

NIH-3T3 cells were seeded at 1.5x10^4^ cells per well in 24-well plates and cultured in complete medium for 6 h to permit adhesion. Cells were then assigned to five groups and treated as follows: (i) control, no treatment; (ii) S.aureus, inoculated with 20 μL of bacterial suspension (108 CFU); (iii) S. aureus /As@EXO, inoculated as in (ii) and supplemented with As@EXO (50 μg/mL); (iv) S. aureus/ KMD@EXO, inoculated as in (ii) and supplemented with KMD@EXO (50 μg/mL); and (v) S. aureus /KMD@AsEXO, inoculated as in (ii) and supplemented with KMD@AsEXO (50 μg/mL). Then the cell was cultured for 18 h, and the cell number was counted by CCK8.


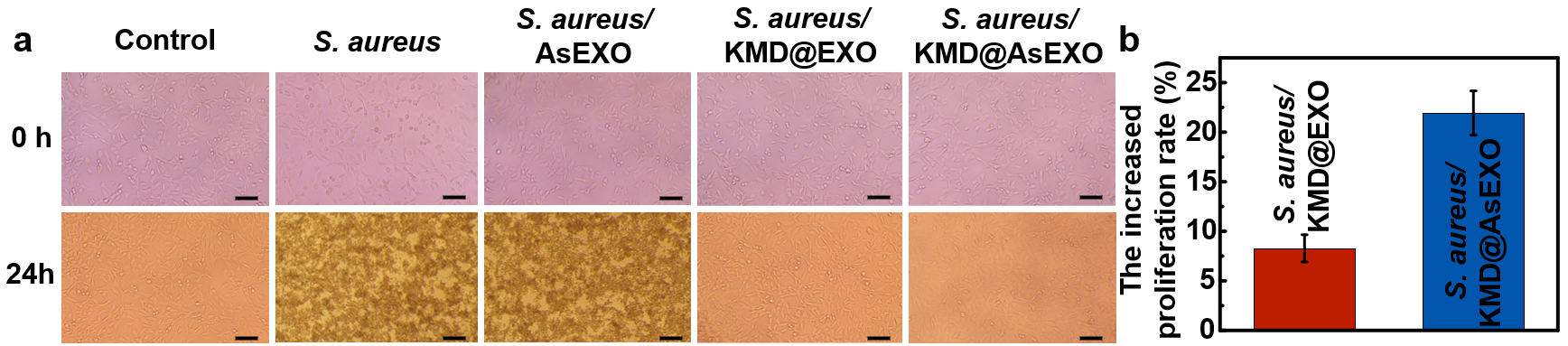


**Figure N3**. Representative micrographs of NIH-3T3 cells infected with *S. aureus* under different treatments (a), and quantification of cell-proliferation rates for the two effective bactericidal treatments relative to the untreated control (b). All scale bars are 100 μm.

As shown in **Figure N3**, exosomes loaded only with asiaticoside and lacking a grafted antimicrobial peptide (*S. aureus*/AsEXO) exhibited no antibacterial activity against *S. aureus*, resulting in contaminated cultures. By contrast, exosomes with a surface-grafted antimicrobial peptide but without internal asiaticoside (*S. aureus*/KMD@EXO) efficiently cleared bacteria but produced only a modest increase in cell proliferation. Notably, only the *S. aureus*/KMD@AsEXO group (combining surface-grafted antimicrobial peptide with internal asiaticoside) both eradicated bacteria and markedly enhanced cellular proliferation, demonstrating a robust synergistic effect.

**Note S4**:

To directly address whether surface loading alters the intrinsic bioactivity of AsEXO, we designed a dose-matched cell-proliferation experiment. We quantified the surface loading of KRWWKWWRRC-PEG-DSPE on KMD@AsEXO and then compared two conditions in NIH-3T3 cells. NIH-3T3 cells were seeded at 9,000 cells per well in 24-well plates and cultured in complete medium for 6 h to allow attachment. Group A received KMD@EXO (50 μg/mL). From mass-balance calculations during KMD@AsEXO preparation, the asiaticoside loading content was ~12.5% and the KRWWKWWRRC-PEG-DSPE grafting ratio was ~8.2%. Accordingly, Group B was treated with the same total mass as Group A, including exosomes, free KRWWKWWRRC-PEG-DSPE, and free asiaticoside. As shown in Figure N4, cell proliferation did not differ significantly between the two groups (P > 0.05), indicating that surface grafting does not compromise the bioactivity of the intravesicular drug.


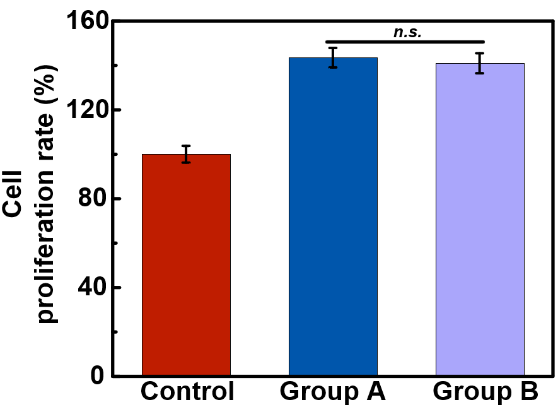


**Figure N4**. The cell proliferation effect of NIH3T3 cells after treatment with KMD@AsEXO and an equal amount of free drugs
